# Systems Biology Analysis of *Zymomonas mobilis* ZM4 Ethanol Stress Responses

**DOI:** 10.1371/journal.pone.0068886

**Published:** 2013-07-16

**Authors:** Shihui Yang, Chongle Pan, Timothy J. Tschaplinski, Gregory B. Hurst, Nancy L. Engle, Wen Zhou, PhuongAn Dam, Ying Xu, Miguel Rodriguez, Lezlee Dice, Courtney M. Johnson, Brian H. Davison, Steven D. Brown

**Affiliations:** 1 Biosciences Division, Oak Ridge National Laboratory, Oak Ridge, Tennessee, United States of America; 2 BioEnergy Science Center, Oak Ridge National Laboratory, Oak Ridge, Tennessee, United States of America; 3 Computer Science and Mathematics Division, Oak Ridge National Laboratory, Oak Ridge, Tennessee, United States of America; 4 Chemical Sciences Division, Oak Ridge National Laboratory, Oak Ridge, Tennessee, United States of America; 5 Department of Biochemistry and Molecular Biology and Institute of Bioinformatics, University of Georgia, Athens, Georgia, United States of America; University Paris South, France

## Abstract

**Background:**

*Zymomonas mobilis* ZM4 is a capable ethanologenic bacterium with high ethanol productivity and ethanol tolerance. Previous studies indicated that several stress-related proteins and changes in the ZM4 membrane lipid composition may contribute to ethanol tolerance. However, the molecular mechanisms of its ethanol stress response have not been elucidated fully.

**Methodology/Principal Findings:**

In this study, ethanol stress responses were investigated using systems biology approaches. Medium supplementation with an initial 47 g/L (6% v/v) ethanol reduced *Z. mobilis* ZM4 glucose consumption, growth rate and ethanol productivity compared to that of untreated controls. A proteomic analysis of early exponential growth identified about one thousand proteins, or approximately 55% of the predicted ZM4 proteome. Proteins related to metabolism and stress response such as chaperones and key regulators were more abundant in the early ethanol stress condition. Transcriptomic studies indicated that the response of ZM4 to ethanol is dynamic, complex and involves many genes from all the different functional categories. Most down-regulated genes were related to translation and ribosome biogenesis, while the ethanol-upregulated genes were mostly related to cellular processes and metabolism. Transcriptomic data were used to update *Z. mobilis* ZM4 operon models. Furthermore, correlations among the transcriptomic, proteomic and metabolic data were examined. Among significantly expressed genes or proteins, we observe higher correlation coefficients when fold-change values are higher.

**Conclusions:**

Our study has provided insights into the responses of *Z. mobilis* to ethanol stress through an integrated “omics” approach for the first time. This systems biology study elucidated key *Z. mobilis* ZM4 metabolites, genes and proteins that form the foundation of its distinctive physiology and its multifaceted response to ethanol stress.

## Introduction

A number of countries around the world have set targets to displace substantial amounts of gasoline with lignocellulosic ethanol (see reviews [1], [2], [3], [4], [5]). *Z. mobilis* are Gram-negative facultative anaerobic ethanologenic bacteria with a number of desirable industrial characteristics, such as unique anaerobic use of the Entner-Doudoroff (ED) pathway that results in low cell mass formation, high-specific productivity and ethanol yield and high ethanol tolerances of up to 85 g/L (11% v/v) for continuous culture and up to 127 g/L (16% v/v) in batch culture [6], [7], [8], [9], [10], [11]. Recombinant strains that utilize pentose sugars such as xylose and arabinose have been developed to overcome the substrate limitations [12], [13].

The *Z. mobilis* ZM4 genome annotation has undergone important improvements [14], and several ZM4 genome scale metabolic models have been derived from it [15], [16]. Genomes for other strains have recently become available and others will be available shortly [17], [18], [19]. *Z. mobilis* recombinant cellulase expression and secretion has been reported [20], and there have been improvements in transformation efficiency by modifying DNA restriction-modification systems [21]. Gene deletions are possible in *Z. mobilis* [22]. DNA microarray studies have provided insights into *Z. mobilis* growth under aerobic and anaerobic conditions [23], under sodium chloride and sodium acetate stress conditions [22], as well as under ethanol and furfural stress [24], [25] that led to insights into its physiology, inhibitor tolerance, and electron transport [26]. Other studies have improved *Z. mobilis* tolerance to different inhibitors using overexpression of the native *hfq* gene [27] or *Deinococcus radiodurans irrE* gene [28].

One of the common stresses that biocatalysts encounter and a bottleneck for bioethanol production improvement is increasing ethanol titers during fermentation. Ethanol is a chaotropic compound that influences membrane stability, as well as the structure and function of macromolecules such as proteins, nucleic acids, and lipids leading to both reduced membrane rigidity and the impairment of metabolic processes [29], [30], [31].

Hopanoids, or bacteriohopanepolyols (BHPs) are sterol substitutes that have been found in a variety of bacteria including *Z. mobilis* and reported to protect against the toxic effects of ethanol [32], [33], [34], [35], [36]. However, there are contradictory reports over the role of hopanoids on ethanol stress tolerance. Moreau *et al.* [37] showed that there were complex changes in the levels of hopanoids and other lipids with the addition of ethanol, but no significant increase in any of the hopanoid lipid classes was identified as ethanol concentration was increased [37]. Other genes have also been reported to be related to ethanol stress response in *Z. mobilis*. For example, alcohol dehydrogenase II was identified as a major stress protein and induced by exposure to ethanol [38], and heat-shock proteins DnaK, GroEL, and GroES increase in abundance after exposure to thermal or ethanol stress, with GroES and GroEL being the two most abundant stress proteins in *Z. mobilis* [39]. Although *Z. mobilis* ethanol responses have been investigated previously, a systematic investigation of *Z. mobilis* ethanol responses using integrated ‘omics’ approaches has not been conducted.

While much progress has been made in understanding *Z. mobilis*, more detailed insights into its physiology and regulatory networks are required to facilitate future metabolic engineering or synthetic biology studies for biocatalyst development. In this study, we combined transcriptomic, proteomic and metabolic profiling with bioinformatic analyses to elucidate the *Z. mobilis* ethanol stress responses and add to the foundation of knowledge on this bacterium.

## Methods

### Bacterial Growth

*Z. mobilis* ZM4 was obtained from the American Type Culture Collection (ATCC31821) and cultured in RM medium (Glucose, 20.0 g/L; Yeast Extract, 10.0 g/L; KH_2_PO_4_, 2.0 g/L, pH5.0) at 30°C as described previously [23]. The growth of *Z. mobilis* under anaerobic conditions was monitored by a Bioscreen C instrument using the 600_nm_ filter (Growth Curves USA, NJ) as described previously [22], [27]. Growth experiments were repeated at least three times and at least two replicates were used for each condition. Duplicate batch fermentations for the ethanol treatment (47 g/L) and control (no added ethanol) conditions were conducted in approximately 2.5-L RM medium in 7.5-L BioFlo110 bioreactors (New Brunswick Scientific, NJ) fitted with agitation, pH, temperature and DOT probes and controls as described previously [23]. Growth was monitored turbidometrically by measuring optical density at 600_nm_ with a model 8453 spectrophotometer (Hewlett-Packard, CA). Samples were harvested during fermentation at different time points, as described previously [23].

### HPLC Analysis of Extracellular Metabolites

High-performance liquid chromatography (HPLC) analysis was used for the measurements of the extracellular metabolite concentration of glucose, acetate, and ethanol in 0.2 µm-filtered samples taken at different time points during fermentation as described previously [23]. Briefly, fermentation samples were acidified with 10 mM sulfuric acid, separated and quantified by HPLC using a LaChrom Elite System (Hitachi High Technologies America, Inc., CA). Analysis was performed with an oven (Model L-2350) set at 60°C, and a pump (Model L-2130) set with a flow rate of 0.5 mL/min in 5 mM H_2_SO_4_. The run time for each sample was set for 35 min (Injector Model L-2200). Eluted compounds were registered and quantified by a refractive index detector (Model L-2490) equipped with a computer-powered integrator. Soluble fermentation products were identified by comparison with retention times and peak areas of corresponding standards. Metabolites were separated on an Aminex HPX-87H, 300×7.8 mm column (Bio-Rad, CA).

### Intracellular Metabolite Analysis by Gas Chromatography-mass Spectrometry (GC-MS)

Culture samples were rapidly pelleted by centrifugation, supernatants removed, cell pellets snap**-**frozen in liquid nitrogen and then stored at –80°C until analysis. A rigorous comparison of sampling approaches for microbial cultures published recently and it was concluded that fast filtering and centrifugation (even at room temperature) produced similar concentrations of metabolites, even for those predicted with high turnover [40]. Metabolite analyses of microbial pellets collected at different time points were similar to that described previously [23]. Cell pellets were suspended with 6–12 mL 80% ethanol (aqueous) with extraction volume proportional to the pellet mass. Cells were disrupted using a sonicator 3000 (Misonix, Inc., NY). An internal standard of 375 µL sorbitol aqueous solution (1 mg/mL) was then added to each tube and 2-mL aliquots were then dried in a helium stream.

Metabolites of two biological samples from each condition were analyzed with a ThermoFisher DSQII GC-MS as trimethylsilyl (TMS) derivatives. The internal standard was added to correct for differences in derivatization efficiency and changes in sample volume during heating and it was not produced by *Z. mobilis* under the assay conditions. Dried exudates were dissolved in 500 µL of silylation–grade acetonitrile followed by the addition of 500 µL N-methyl-N-trimethylsilyltrifluoroacetamide (MSTFA) with 1% trimethylchlorosilane (TMCS) (Pierce Chemical Co., Rockford, IL), and samples then heated for 1 h at 70°C to generate. After 4 days, 1-µL aliquots were injected into the GC-MS, fitted with an Rtx-5MS (crosslinked 5% PH ME Siloxane) 30 m×0.25 mm×0.25 µm film thickness capillary column (Restek, Bellefonte, PA). The standard quadrupole GC-MS was operated in electron impact (70 eV) ionization mode, with 6 full-spectrum (70–650 Da) scans per second. Gas (helium) flow was set at 1.1 mL per minute with the injection port configured in the splitless mode. The injection port and detector temperatures were set to 220°C and 300°C, respectively. The initial oven temperature was held at 50°C for 2 min and was programmed to increase at 20°C per min to 325°C and held for another 11.25 min, before cycling back to the initial conditions. Quantified metabolites of interest were extracted using a key selected m/z that was characteristic for each metabolite, rather than the total ion chromatogram, to minimize integration of co-eluting metabolites. Peaks were quantified by area integration and the concentrations were normalized to the quantity of the internal standard (sorbitol) recovered, amount of sample extracted, derivitized, and injected. Metabolite data of ZM4 under ethanol control and treatment conditions were averaged and presented as relative responses between ZM4 under ethanol treatment fermentation versus ZM4 without ethanol treatment. Treatment differences between ethanol treated versus control cultures at a given sample collection time were analyzed by Student’s t-tests with differences considered significant at P≤0.05.

### Microarray Analysis and Validation

Microarray analysis was conducted essentially as described previously [22]. Briefly, samples were harvested by centrifugation and the TRIzol reagent (Invitrogen, Carlsbad, CA) was used to extract total cellular RNA. Each total RNA preparation was treated with RNase-free DNase I (Ambion, Austin, TX) to digest residual chromosomal DNA and subsequently purified with the Qiagen RNeasy Mini kit in accordance with the instructions from the manufacturer. Total cellular RNA was quantified at OD_260nm_ and OD_280nm_ with a NanoDrop ND-1000 spectrophotometer (NanoDrop Technologies, DE) and the RNA quality was checked with Agilent Bioanalyzer (Agilent, CA). High-quality, purified total RNA was used as the template to generate ds-cDNA using Invitrogen ds-cDNA synthesis kit (Invitrogen, CA). The ds-cDNA was sent to NimbleGen for labeling, hybridization, and scanning following the company's protocols. Quality assessments, normalization and statistical analyses were conducted using JMP Genomics 4.0 software (SAS Institute, Cary, NC) as described previously [23], except that DNA microarray data were normalized using the LOWESS method [41]. An analysis of variance (ANOVA) statistical test determined differential expression levels between conditions and time points using the False Discovery Rate testing method. Differences were considered significant at P≤0.05 and comparisons were also conducted using a two-fold filtering criterion [42]. The expression profiles generated in this study have been deposited in GEO database under the accession number GSE21165 so that others may access the data and apply their own testing methods and criteria. The interaction among ethanol-regulated genes was investigated using the String 8.2 pre-computed database [43], available at http://string.embl.de/.

Microarray data were validated using real-time qPCR as described previously [22], [23], except that the Bio-Rad MyiQ2 Two-Color Real-Time PCR Detection System (Bio-Rad, CA) and Roche FastStart SYBR Green Master (Roche Applied Science, IN) were used for this experiment. Ten genes representing different functional categories and a range of gene expression values based on microarray hybridizations were analyzed using qPCR from cDNA derived from different time point samples. Primer pairs were designed as described previously, and the oligonucleotide sequences of the 10 genes selected for qPCR analysis are listed (file S1).

### Proteomic Analysis

Shotgun proteomic measurements were carried out using the MudPIT approach [44]. Briefly, proteins were extracted from fermentation culture cell pellets by sonication and centrifugation. Two ethanol-treated cultures were examined, as were two control cultures. Total protein concentrations were then quantified with the Bio-Rad DC Protein Assay Kit II (Bio-Rad, CA). Proteins were digested by trypsin after denaturing and reducing with 6 M guanidine and 10 mM dithiothreitol (DTT) (Sigma Chemical Co., MO). Digested proteins were then reduced with 20 mM DTT and desalted using C18 solid-phase extraction (Sep-Pak Plus, Waters, MA). The protein digests were loaded on split-phase back columns containing reverse-phase and strong cation exchange phases [45]. Two-dimensional LC separation was performed with twelve salt pulses of increasing strength each of which was followed by a 2-h reverse-phase gradient. MS/MS analysis was performed on an LTQ linear ion trap mass spectrometer (Thermo Scientific, CA) with dynamic exclusion enabled (repeat count  = 1; exclusion duration  = 180 s).

MS/MS spectra were extracted into MS2 files [46] from RAW files using the program Raw2MS2, version 1.0, which was developed locally [47], [48]. All MS/MS scans were searched against the FASTA database containing all 1,946 reannotated *Z. mobilis* proteins [14]) and their reversed sequences, with a total of 3,892 protein entries using the SEQUEST program (version 27) [49], with mass tolerance for precursor ions  = 3.0 and mass tolerance for fragment ions  = 0.5. No peptide modifications were specified. Peptide identifications were filtered and assembled into proteins by the DTASelect program (version 1.9) [50] using its default criteria (at least two fully tryptic peptides, up to 4 missed cleavages, a delCN of at least 0.08 and cross-correlation scores (Xcorrs) of at least 1.8 (for charge state [z]  = +1), 2.5 (z = +2), and 3.5 (z = +3). The DTASelect parameter (*−p 2*) that requires two peptides also permits protein identifications arising from a single peptide sequence for which MS-MS spectra are identified from parent ions of two or more change states; these identifications were subsequently removed from the reported results. The false discovery rate for peptide identification was ≤∼1% when using these default parameters [51]. The isoelectric point and molecular mass distributions were generated for the predicted proteome using the Expasy pI and molecular weight tool (http://ca.expasy.org/tools/pi_tool.html).

To estimate relative abundance differences of proteins, each spectrum count value was increased by 1 to avoid division by zero errors [52]. These adjusted spectrum count values were normalized such that the sum of adjusted spectrum count values for each individual MudPIT analysis was equal to the average of this quantity across all four analyses. Spectrum count values from MudPIT analyses of duplicate cultures of ethanol treated cells were pooled, as were results for the control cells. Identification of protein abundance changes between ethanol-treated versus control cells was based on a G test applied to the adjusted and normalized spectrum count values [52], [53]. A protein was determined to be different in abundance in the ethanol-treatment condition if the p value resulting from comparison of the G statistic with a χ^2^ distribution (one degree of freedom) was less than 0.05 and the normalized spectrum count for both biological replicates from ethanol treatment condition were higher (or lower) than those from the control condition. Additionally, for a given protein, the p value resulting from the G test, pj, was required to pass the Benjamini-Hochberg criterion such that pj≤q(j/t), where q is the chosen false discovery rate (set at 0.05), t is the total number of identified proteins, and j is the rank of that protein in a list ordered by increasing p value [53]. Proteins passing these criteria are denoted as “UP” or “DOWN” while those not passing the criteria are denoted as “NULL.” To estimate the rate of false discovery for proteins with increased or decreased abundance, the G test was applied to the two replicates of ethanol-treated cells, and also to the two replicates of the control cells [54]. Identified peptides with sequences that are found in more than one *Z. mobilis* protein are identified in Table S2 in file S2, and the corresponding proteins containing these peptides are listed in Table S3 in file S2. The data associated with this manuscript may be downloaded from the ProteomeCommons.org Tranche network using the following hash:/29rG1vBesn7r/1UyybgeueybqrFf3P63Ipz8PzhfZD/dWurBeqrJ5ajE6eSQBQmfmAI+it0t9x+XFI8qJwhal0+72EAAAAAAAAw7w =  = .

### Operon Prediction

Operons were predicted based on a previously published method [55], [56], [57]. In brief, a classifier was trained based on experimentally validated operons of *E. coli* and *B. subtilis*. The classifier was tested on five other genomes with limited number of operons available, and it was shown that the accuracy was comparable to the training data sets. The classifier was then applied to *Z. mobilis* genome without further modification.

### Operon Adjustment through Microarray Data Analysis

Note that an adjacent gene pair could be in either two consecutive operons, hence called a boundary pair, or the same operon, called an operon pair. From previous studies, we found that adjacent gene pairs with prediction-score less than 1.5 and larger than 5 have very high confidence of being operon pairs and boundary pairs, respectively [55], [56], [57]. As a result, only those adjacent gene pairs with prediction-score between 1.5 and 5 were adjusted using microarray data. The microarray data underwent a discretization transformation. Briefly, two arrays (duplicated experiments) of expression values for ZM4 wild-type growth without ethanol stress at the time of 6 hours were used to calculate the gene expression base value for each gene in the data. Then, if the difference between the expression value and the base value falls into [−0.5, 0.5), the discretized value is set to be 0; and [0.5, 1.5) to 1; [1.5, 2.5) to 2; [2.5, 3.5) to 3; [3.5, ∞) to 4; and similarly for the down-regulated expression values. These discretization cutoffs were determined as described previously [58], to maximize the correlation of the identified expression patterns with predicted operon structure. After that, the discretized microarray data were represented using a matrix of integers between −4 and 4 to represent their gene expression levels in the corresponding experiments. For each adjacent gene pair, their *microarray-score* is assigned with the maximum number of experiments under which they are at the same expression levels. Then, the adjusted prediction-score was calculated as followed: AdjpredictionScore = predictionScore + ln (f_bp_(X)/f_op_(X)), where f_bp_ and f_op_ are the frequencies of the boundary and operon pairs with the same microarray-score X, respectively. In this work, the ethanol stress microarray data generated in this study (GSE21165) and the microarray data from a previous salt stress study (GSE18106) [22] were used. These two microarray studies were conducted using same procedure, platform and protocols. Raw data from the two experiments were combined and processed together using JMP Genomics 4.0 software using LOWESS normalization method as described above.

## Results

### Physiological Response to Ethanol

**Figure 1:**
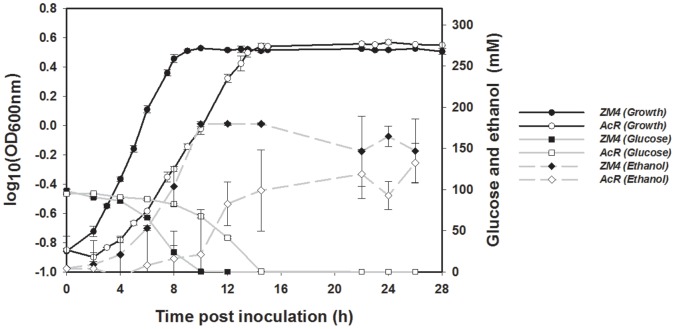
The growth, glucose consumption and net ethanol production of *Z. mobilis* in the absence or presence of 47 g/L (or 6% [v/v]) ethanol. Black and grey arrows indicate the sampling time points of transcriptomic and metabolomic studies for *Z. mobilis* in the absence of ethanol (control, black arrows) or in the presence of ethanol (treatment, grey arrows) respectively. The exponential phase samples (control at 6 h and treatment at 10 h post-inoculation) were used for proteomic study.

The growth of *Z. mobilis* supplemented with 0, 16, 32, 47, 63, 79, 95 or 118 g/L ethanol was assessed using a Bioscreen C instrument under anaerobic conditions to determine an appropriate ethanol concentration for systems biology studies. *Z. mobilis* growth was arrested when ethanol was added to the medium at or above 79 g/L, and minor differences were observed between untreated control cultures and where ethanol was added at concentrations of 16 and 32 g/L (Fig. S1 in file S5). *Z. mobilis* showed intermediate growth rate decreases when rich medium (RM) cultures were amended with 47 g/L (6% v/v) ethanol, and cultures treated with this concentration of ethanol attained final cell densities similar to untreated control cultures (Fig. S1 in file S5). The concentration of 47 g/L ethanol was chosen for the treatment in the subsequent systems biology study to impact cell growth slightly (Fig. 1). For simplicity in the present manuscript, control refers to no ethanol supplementation; and ethanol treatment refers to the supplementation of 47 g/L ethanol; unless otherwise stated, the absolute ethanol concentration is omitted.

The presence of ethanol negatively affected growth in *Z. mobilis* ZM4 fermentations (Fig. 1). *Z. mobilis* had a maximal culture density of 7.0 OD_600nm_ units approximately 9 h post-inoculation without ethanol supplementation, while *Z. mobilis* only reached its highest culture density of 6.5 OD_600nm_ units at 13 h post-inoculation with the supplementation of 47 g/L ethanol despite initial inocula concentrations being slightly greater than that of control condition (Fig. 1). The growth rate of ethanol-treated cells was reduced by more than one third compared to that of control cells, dropping from 0.47±0.008 hour^−1^ to 0.31±0.01 hour^−1^. For comparison of equivalent exponential phase growth, *Z. mobilis* control at 6 h and ethanol-treated cells at 10 h (ETOH_10h/Control_6h) were usually used for comparison purposes, unless otherwise described. In addition, the early stationary phase (transition from exponential phase to stationary phase) comparison was for the ethanol-treated cell at 13.5 h to control at 10 h (ETOH_13.5h/Control_10h), and the late stationary phase comparison was of ethanol-treated cells at 26 h to control at 26 h (ETOH_26h/Control_26h).

HPLC was used to quantify and compare *Z. mobilis* glucose consumption and the kinetics of ethanol, lactate, and acetate production either with or without added ethanol. *Z. mobilis* consumed glucose more slowly with ethanol supplementation; more than 71% of the initial glucose was not utilized by *Z. mobilis* at 10 h post-inoculation, and an additional 4.5 h was needed to fully utilize the remaining glucose (Fig. 1; file S3). In contrast, only 2.9% of the initial glucose concentration (98 mM or 0.053 g/L) remained at 10 h post-inoculation in the absence of added ethanol (Fig. 1; file S3). *Z. mobilis* ethanol production was correlated with substrate (glucose) consumption. Where growth was reduced with supplemental ethanol, slower ethanol production was seen compared to control cells (Fig. 1; file S3). In addition, ethanol-treated *Z. mobilis* had a reduced net ethanol production with a highest net ethanol production of 6.1 g/L compared to that of 8.3 g/L for control cells (Fig. 1; file S3). Similarly, acetate production was reduced dramatically from 0.121±0.002 g/L to 0.054±0.004 g/L with ethanol supplementation at 26 h post-inoculation. The majority of lactate was produced after exponential growth and in contrast with ethanol and acetate production, lactate production in ethanol-treated *Z. mobilis* increased about one-fifth to 0.046±0.001 g/L after 14.5 h post inoculation compared to that of control, and remained relatively steady through the end of the experiment (file S3).

### Metabolomics of *Z. mobilis* in Response to Ethanol

The physiological status of *Z. mobilis* was investigated further by a comparative GC-MS analysis of intracellular metabolomic profiles that examined relative differences at different time points post-inoculation (6, 10, 12, 13.5 or 26 h). Metabolite identification and analysis gave 45 metabolites that were present at different abundance levels between *Z. mobilis* with and without ethanol treatment (file S4). Fewer metabolites had at least a 1.5-fold or greater change during exponential phase growth compared to that of earlier stationary or stationary phase comparisons (file S4). In ethanol-treated cells, most observed metabolites accumulated in the early time point samples and treatment differences then diminished thereafter. This may be either the ethanol effect slowing a pathway or a transient response to overcome ethanol toxicity. Tryptophan increased over time in the control, ethanol treated microbes had only 3% of the tryptophan levels of the controls in the later two sampling time points. Of the metabolites quantified, tryptophan was the most impacted by ethanol stress. Transcriptomic analysis indicated the upregulation of genes for tryptophan synthesis (ZMO0584-7, which forms an operon with two other genes of ZMO0582 and ZMO0583), however; when all variables were taken into consideration together the changes were less than the cutoff value of at least 2 fold (Table S7, Table S13 in file S2).

Previous studies showed that the lipid membrane is related to *Z. mobilis* ethanol stress [32], [33], [34], [35]. In this study, we identified several metabolites related to lipid metabolism, such as glyceric acid, palmitic acid, and stearic acid, which were present at elevated levels in the ethanol-stressed *Z. mobilis* compared to those of the control cells at 10 h but not at other sampling points (file S4). They accumulated in the early stationary phase in response to ethanol treatment, but treatment differences were again absent in the stationary phase, although the relative amount of fatty acids was only a small fraction of the total metabolites identified (file S4). Similarly hopanoids were detected via GC-MS, but their levels were relatively low and not considered significantly different between the control and the ethanol stress condition in the present experiment (data not shown).

Hirasawa et al. found that the addition of tryptophan to the culture medium, over-expression of tryptophan permease gene or tryptophan biosynthesis genes increased the ethanol stress tolerance [59]. Based on our results (file S4) and previous studies, we investigated whether or not the supplementation of glycerol or tryptophan in the fermentation media could improve *Z. mobilis* ethanol tolerance. While the addition of supplemental tryptophan did not appear to have an effect on ethanol tolerance (data not shown), medium supplementation with glycerol at low concentrations (0.001%) improved *Z. mobilis* growth marginally as it entered stationary phase under ethanol stress conditions on a consistent basis but not at levels considered significant in this study (Fig. S2 in file S5).

### Proteomic Profiling of *Z. mobilis* in Response to Ethanol

Shotgun proteomics was used to compare exponentially growing *Z. mobilis* cells. To reduce the growth-phase effect on protein expression difference, *Z. mobilis* cells at either 10 h post-inoculation for treatment or at 6 h post inoculation for control cells were at a similar mid-exponential phase, and therefore used for comparison (Fig. 1). We identified 942 proteins, which is about 55% of the total predicted *Z. mobilis* ZM4 proteins (Table S6 in file S2 **)**. The isoelectric point and molecular mass distributions for the identified proteins were similar to the corresponding distributions for all proteins in the revised genome annotation of *Z. mobilis* [14] (Fig. S3 in file S5). This is the largest number of expressed proteins described for *Z. mobilis* to date (Table S2, Table S6 in file S2).

**Figure 2:**
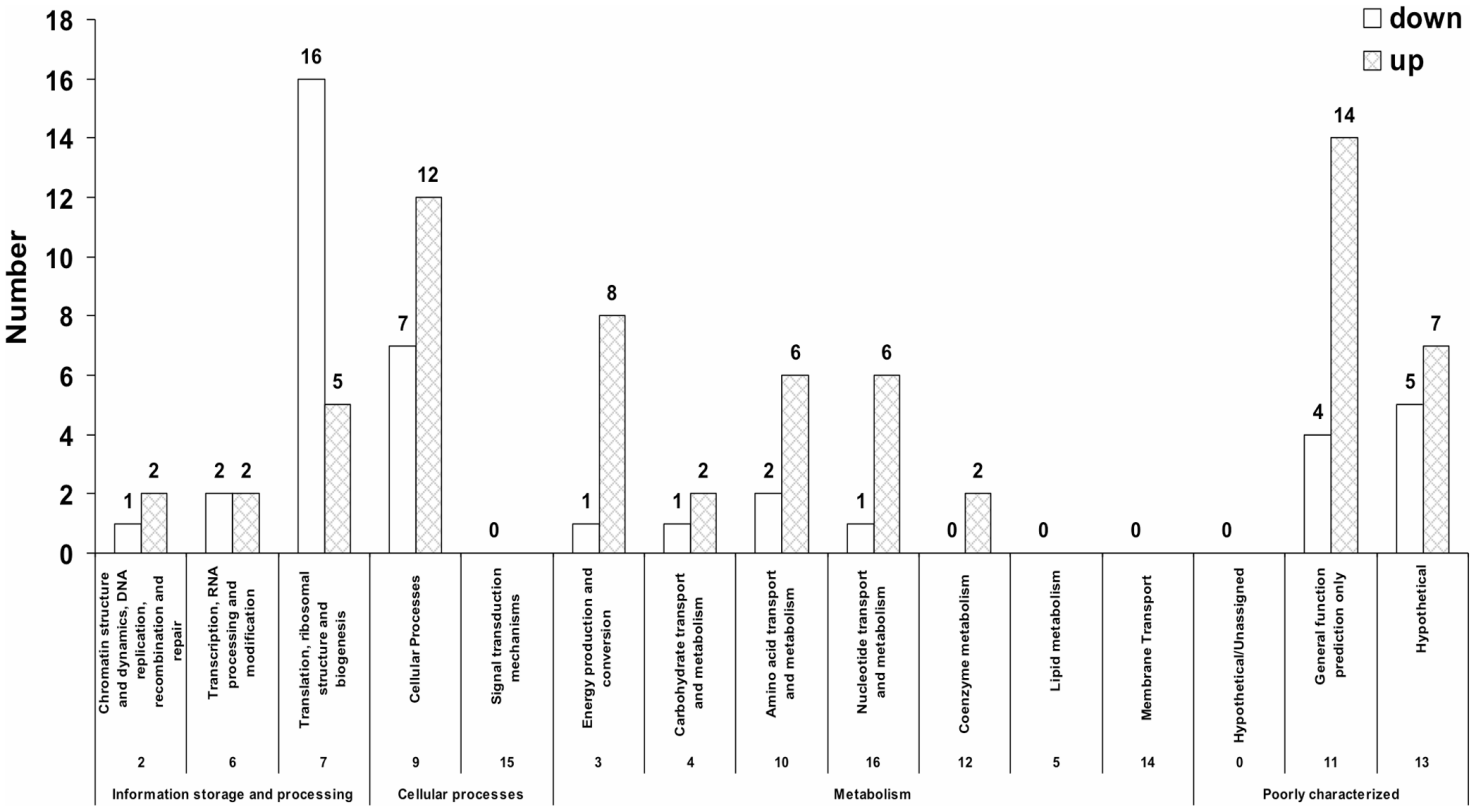
Functional categories of the eighty-four ethanol differentially regulated proteins with at least 1.5-fold significant changes at exponential phase for 47 g/L ethanol treated cells at 10 h versus control cells at 6 h. The functional categories are based on COG (Clusters of Orthologous Groups of proteins) categories.

**Figure 3:**
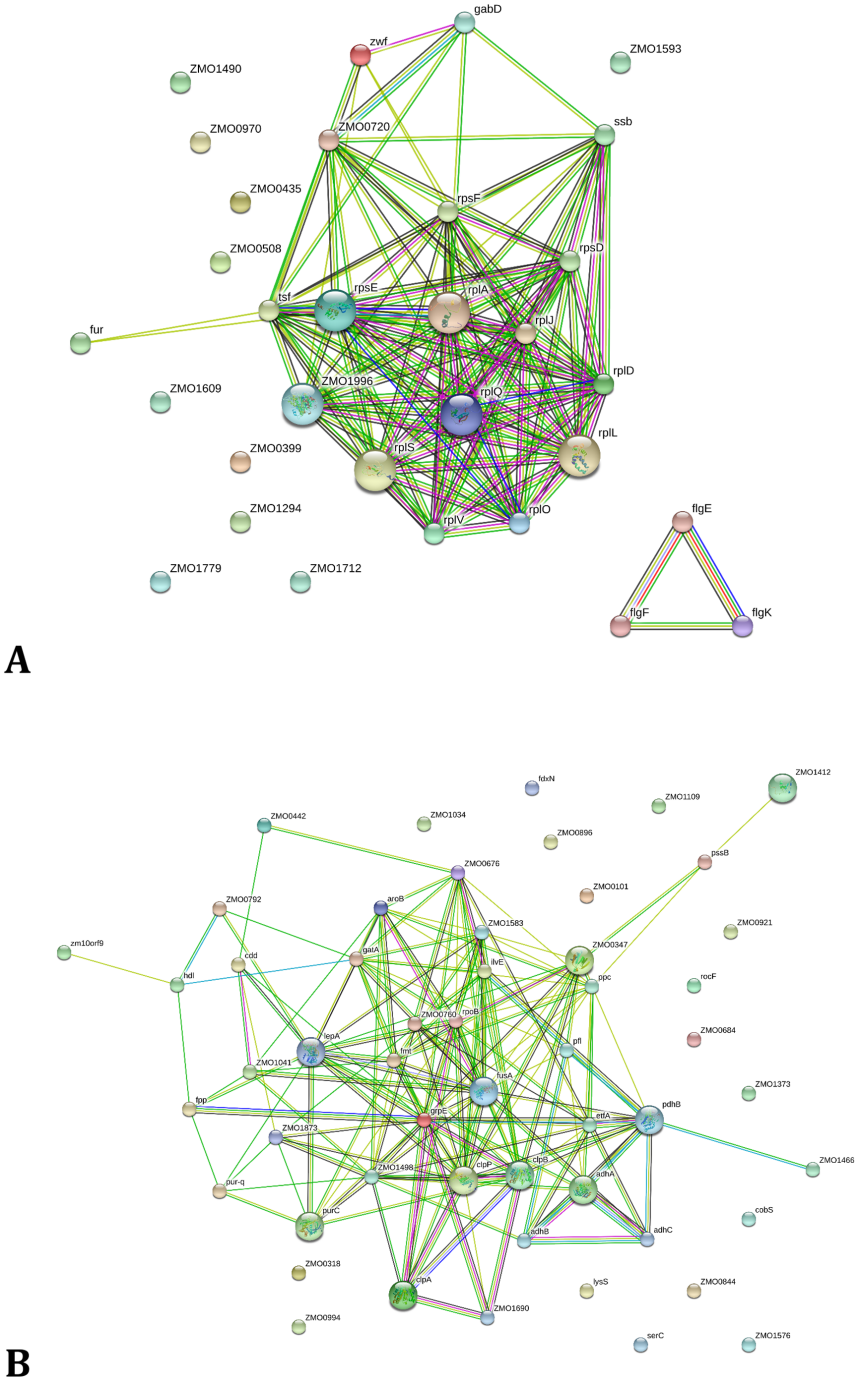
Documented interactions based on String 8.2 database for 31 ethanol down-regulated proteins (A) and 53 ethanol up-regulated proteins (B) with at least 1.5-fold changes from proteomic study. A greater the number of lines associated with the connection, indicates a greater level of confidence in the association. The network nodes are proteins. The edges represent the predicted functional associations. An edge may be drawn with up to 7 differently colored lines - these lines represent the existence of the seven types of evidence used in predicting the associations. A red line indicates the presence of fusion evidence; a green line - neighborhood evidence; a blue line - coocurrence evidence; a purple line - experimental evidence; a yellow line - textmining evidence; a light blue line - database evidence; a black line - coexpression evidence.

We identified 95 differentially detected proteins based on a comparison of spectrum count values (i.e., the number of tandem mass spectra that identify tryptic peptides of a particular protein) [52], [53], [54], of which 84 had at least a 1.5-fold change and 61 had a 2-fold or higher difference (Table 1 and Table S6 in file S2). Two differentially detected proteins (ZMO0593 and ZMO0734) contain peptides that also could be assigned to other *Z. mobilis* proteins; Table S3 in file S2 lists the 30 identified proteins that contain non-unique peptides. To estimate the rate of false discovery of differential proteins, we also applied the G test [54] for comparing the two ethanol-treated biological replicate samples to each other, as well as the two control samples, leading to identification of much lower numbers (8 and 20, respectively) of differentially detected proteins. We identified 53 up-regulated proteins and 31 down-regulated proteins for the ethanol treatment at exponential phase compared to the untreated control in the same growth phase at the 1.5-fold cutoff (Table S6 in file S2), and these were sub-grouped by their COG (Clusters of Orthologous Groups of proteins) functional categories [60] separately (Fig. 2 and Table S6 in file S2 **)**. We present the proteins that showed two-fold significant changes (Table 1). Approximately two-fifths of the down-regulated proteins belong to the functional category of translation, ribosomal structure and biogenesis. About one-third of the up-regulated proteins are within the metabolism category, and most are related to energy production/conversion, as well as nucleotide transport proteins (Fig. 2 and Table S6 in file S2). The interactions among the down-regulated or up-regulated proteins with at least a 1.5-fold change were also analyzed for their previously documented interactions using STRING database [43] (Fig. 3 **)**. Among the 31 ethanol down-regulated proteins with at least 1.5-fold change, ribosomal proteins as well as proteins involved in flagellar synthesis and DNA replication have been shown to interact with each other (Fig. 3A and Table S6 in file S2). The 53 ethanol up-regulated proteins with at least 1.5-fold change had fewer interactions; chaperone proteins, DNA and protein repair proteins, as well as alcohol dehydrogenase and proteins involved in the ED pathway and energy metabolism showed stronger linkages than the rest of the ethanol up-regulated proteins (Fig. 3A and Table S6 in file S2).

**Table 1:** Comparison of array data to significantly different proteins.

			Proteomics		Array	
Gene	Product	Ratio^a^	p-value	Ratio	p-value	Sig Index^b^
ZMO1373	TIR protein	3.4	9.50E-06	0.7	5.13E-08	1
ZMO1818	4Fe-4S ferredoxin iron-sulfur binding domain-containing protein	3.2	8.40E-05	−0.3	3.89E-03	1
ZMO0101	putative oxidoreductase	3.2	9.60E-05	1.5	1.66E-31	1
ZMO0405	ATP-dependent Clp protease, ATP-binding subunit ClpA	3.1	2.00E-04	−0.7	5.37E-14	1
ZMO0347	RNA-binding protein Hfq	3.0	3.10E-04	−0.1	5.37E-01	0
ZMO0844	sporulation domain-containing protein	2.9	8.60E-04	0.7	1.05E-06	1
ZMO0896	hypothetical protein	2.9	8.60E-04	0.5	2.40E-12	1
ZMO1041	NUDIX hydrolase	2.8	1.20E-03	−1.2	1.17E-13	1
ZMO0864	cytidine deaminase	2.8	1.60E-03	0.3	1.91E-07	1
ZMO0760	lactoylglutathione lyase	2.8	1.80E-03	0.0	7.08E-01	0
ZMO1466	alpha/beta family hydrolase	2.7	1.10E-05	0.4	1.48E-16	1
ZMO1583	DNA topoisomerase (ATP-hydrolyzing)	2.6	3.40E-03	0.1	1.74E-02	1
ZMO1370	nitrilase/cyanide hydratase and apolipoprotein N-acyltransferase	2.6	4.80E-03	−0.4	7.08E-11	1
ZMO1722	S-(hydroxymethyl)glutathione dehydrogenase/class IIIalcohol dehydrogenase	2.6	4.80E-03	0.3	6.92E-09	1
ZMO1544	Cobaltochelatase	2.6	4.50E-05	0.5	2.75E-15	1
ZMO1576	putative short-chain dehydrogenase/oxidoreductase	2.5	1.80E-05	−0.2	9.55E-05	1
ZMO1605	pyruvate dehydrogenase subunit beta	2.3	4.10E-06	1.0	5.62E-23	1
ZMO1741	GTP-binding protein LepA	2.0	4.90E-03	0.4	1.62E-08	1
ZMO0811	methionyl-tRNA formyltransferase	1.9	2.20E-03	−0.1	1.91E-02	1
ZMO0734	3'(2'),5'-bisphosphate nucleotidase	1.9	3.80E-04	0.2	1.66E-04	1
ZMO1412	MucR family transcriptional regulator	1.9	2.90E-03	0.9	1.66E-20	1
ZMO1873	glutaredoxin-related protein	1.8	5.40E-03	0.5	3.31E-12	1
ZMO1052	Phosphoribosylaminoimidazolesuccinocarboxamide synthase	1.8	2.30E-03	1.2	1.23E-14	1
ZMO0442	HAD-superfamily hydrolase, subfamily IA, variant 3	1.7	3.20E-03	−0.1	6.31E-01	0
ZMO1334	YceI family protein	1.7	4.30E-05	1.4	3.98E-18	1
ZMO0318	short-chain dehydrogenase/reductase SDR	1.7	2.80E-08	−1.1	8.51E-26	1
ZMO0948	Endopeptidase Clp	1.7	6.30E-08	0.6	3.47E-14	1
ZMO1424	ATPase	1.7	1.20E-11	0.5	3.31E-05	1
ZMO0921	hypothetical protein	1.7	1.20E-03	0.3	1.55E-13	1
ZMO0913	branched-chain amino acid aminotransferase	1.6	5.50E-03	0.7	2.09E-16	1
ZMO0432	arginase family protein	1.5	6.20E-04	0.9	6.17E-26	1
ZMO1684	Phosphoserine transaminase	1.4	1.90E-05	0.8	4.57E-26	1
ZMO0016	GrpE protein	1.3	7.30E-06	0.6	1.17E-12	1
ZMO0855	farnesyl-diphosphate synthase	1.3	2.30E-05	0.3	4.79E-10	1
ZMO1690	heat shock protein DnaJ domain-containing protein	1.2	1.20E-03	−0.8	6.17E-21	1
ZMO1570	Formate C-acetyltransferase	1.2	4.40E-04	0.3	5.89E-04	1
ZMO0593	3-dehydroquinate synthase	1.1	4.70E-04	0.4	6.61E-21	1
ZMO1498	histidine triad (HIT) protein	1.1	2.50E-03	0.0	6.92E-01	0
ZMO0684	CRISPR-associated Csy3 family protein	1.1	1.90E-04	0.8	2.69E-10	1
ZMO1496	Phosphoenolpyruvate carboxylase	1.1	2.30E-05	0.2	2.63E-04	1
ZMO0792	dihydroorotase	1.0	2.80E-03	0.4	1.00E-09	1
ZMO1034	calcium-binding EF-hand-containing protein	1.0	2.20E-06	−0.3	2.69E-02	1
ZMO1294	sugar isomerase (SIS)	−3.5	2.00E-05	−0.1	5.50E-01	0
ZMO1593	peptidase M61 domain-containing protein	−2.9	2.40E-03	−0.1	6.92E-02	0
ZMO0508	GCN5-related N-acetyltransferase	−2.7	4.60E-03	−0.1	1.91E-01	0
ZMO0399	hypothetical protein	−2.1	2.90E-04	−0.1	5.25E-01	0
ZMO0970	putative purine nucleoside permease	−2.1	1.30E-03	0.3	9.55E-06	1
ZMO0610	flagellar basal-body rod FlgF	−1.8	2.00E-04	0.4	1.15E-03	1
ZMO0605	flagellar hook-associated protein FlgK	−1.8	1.10E-03	0.0	8.32E-01	0
ZMO1411	ferric uptake regulator family protein	−1.4	1.90E-03	0.2	2.14E-03	1
ZMO0611	flagellar basal body FlaE domain-containing protein	−1.4	5.10E-18	0.2	1.48E-02	1
ZMO1609	hypothetical protein	−1.3	9.60E-08	0.0	6.61E-01	0
ZMO1542	single-strand binding protein	−1.2	2.60E-04	−0.8	7.76E-08	1
ZMO1712	FKBP-type peptidyl-prolyl cis-trans isomerase 1-like	−1.2	6.70E-05	0.0	7.76E-01	0
ZMO0727	50S ribosomal protein L10	−1.1	3.10E-13	−0.1	8.91E-03	1
ZMO0518	50S ribosomal protein L4	−1.1	2.30E-15	0.3	3.31E-03	1
ZMO1779	hypothetical protein	−1.1	1.60E-17	−0.2	1.35E-05	1
ZMO1490	hypothetical protein	−1.1	4.90E-04	−0.7	1.12E-11	1
ZMO0533	30S ribosomal protein S5	−1.1	2.80E-15	0.8	1.41E-22	1
ZMO1079	50S ribosomal protein L19	−1.1	1.40E-06	−0.3	5.50E-04	1
ZMO0542	50S ribosomal protein L17	−1.0	1.90E-07	0.4	1.15E-02	1

a Ratios are represented as log2 values for 10 h treatment condition over 6 h control condition.

b Significance index shows if a gene was significantly differentially expressed (1) or not (0) for this comparison.

Among proteins with highest normalized spectrum counts, and therefore likely to be abundant [61], were ribosomal proteins, enzymes involved in the ED pathway, and chaperones (Table S6 in file S2). Ribosomal proteins (ZMO0726, ZMO0728) and translation elongation factor Ts (ZMO1155) were the most abundant proteins down-regulated by ethanol treatment. Cystathione gamma-synthase (ZMO0676), iron-containing alcohol dehydrogenase (ZMO1596) and a hypothetical protein (ZMO1109) were among the most abundant up-regulated proteins. Enzymes that were abundant in both the ethanol-treated and control conditions included key enzymes such as pyruvate kinase (Pyk, ZMO0152), phosphopyruvate hydratase (Eno, ZMO1608), and glyceraldehyde-3-phosphate dehydrogenase (Gap, ZMO0177); as well as proteins like chaperone DnaK (ZMO0660) and chaperonin GroEL (ZMO1929)(Table S6 in file S2).

### Transcriptomic Profiling of *Z. mobilis* in Response to Ethanol

**Figure 4:**
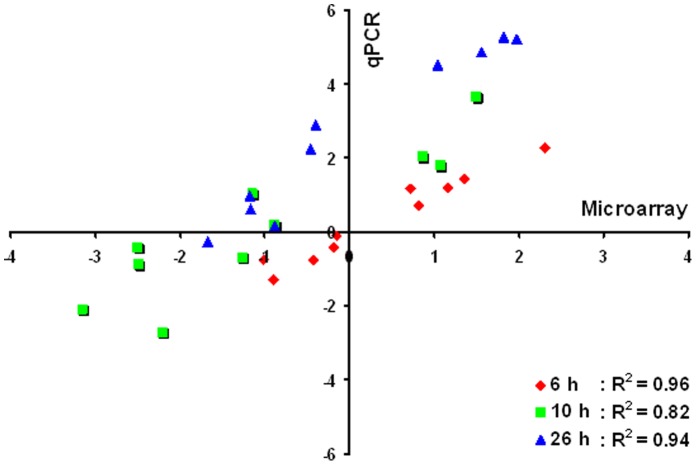
Correlation between microarray and RT-qPCR results for microarray data verification. Comparison of gene expression measurements by microarray and qRT-PCR between wild-type *Z. mobilis* ZM4 with the treatment of 47 g/L ethanol and control cells without ethanol treatment at different time points. The gene expression ratios of both microarray data and qPCR data for ten genes were log transformed in base 2 (log_2_<Ethanol_treatment/Control>), and the microarray log2 ratio values were plotted against the qRT-PCR log2 values.

Samples from the control fermentation and the ethanol-treated fermentation were taken at 6, 10, 13.5, and 26 h post-inoculation and the gene expression profiles were analyzed using a NimbleGen high density expression array as described previously [22]. The microarray data were assessed using several approaches and the quality was found to be high. The correlation coefficients for microarray data from biological replicates were good, with r >0.98 for each comparison (Fig. S4A in file S5). In addition, the microarray data of biological replicates were also grouped together closely based on the hierarchical clustering and principal components analyses (Fig. S4B–C in file S5), and that data can be grouped or separated as time points advance through the experiment and also by treatment before the application of the ANOVA testing. Finally, ten differentially expressed genes from different functional categories with a broad range of expression ratios were chosen for real-time quantitative PCR (RT-qPCR) validation (Table S1). RT-qPCR results indicated a high degree of concordance between microarray and RT-qPCR data (Fig. 4).

In the absence of any fold-change filtering, about seventeen hundred genes were identified to be significantly differentially expressed using the ANOVA with treatment and time as variables (Table S7 in file S2), covering nearly all of the *Z. mobilis* ZM4 genes reannotated by our group [14] indicating a dynamic gene expression for ethanol responses. Two comparisons were then carried out to identify the ethanol-responsive genes, by comparing the significant genes between the ethanol-treated cells and the control cells at different growth phases, as well as by comparing the time series of differentially expressed genes within ethanol-treated cells (Fig. S5 in file S5). In addition, the identification of differentially expressed genes in the time series study of control cells helps exclude growth-phase related genes. Differentially expressed genes identified from both the treatment versus control study and the ethanol-treatment time series study but not from the control time series study were regarded as ethanol-responsive genes (Fig. S5 in file S5).

By comparing gene expression between ethanol-treated and control cells at the same growth phase, 483 genes were significant with at least a 2-fold change (Fig. S5 in file S5, Table S8 in file S2). About 10% of the 483 genes showed differential gene expression during an exponential growth comparison. The majority of differentially expressed genes responded to the transition from exponential phase to stationary phase (Fig. S5 in file S5, Table S8 in file S2). Similar to proteomic data (Table 1), ethanol treatment repressed the expression of genes related to the function of information storage and processing, with more genes associated with cellular processes being up-regulated in response to the treatment (Table S8 in file S2). Five genes were constitutively down-regulated with the ethanol treatment, which are a subfamily IA HAD-superfamily hydrolase (ZMO1862), Beta-fructofuranosidase (ZMO0375), exodeoxyribonuclease III Xth (ZMO1699), DNA primase (ZMO1622), and a hypothetical protein (ZMO0930) (Table S8 in file S2). Only two genes were constitutively ethanol upregulated, namely a putative oxidoreductase (ZMO0101) and an YceI family protein (ZMO1334) (Table S8 in file S2).

With a cut-off value of at least a 2-fold change, a time series comparison identified 571 genes significantly expressed in ethanol-treated cells (Fig. S5 in file S5, Table S9 in file S2). 112 genes were identified to be related to ethanol stress but not growth phase (Table S10 in file S2). The time series gene expression of ethanol-treated cells had a different pattern of functional categories from the treatment versus control comparison, with more genes related to cellular processes down-regulated and more genes associated with metabolism and information storage and processing up-regulated (Table S10 in file S2).

Thirty-four genes were identified to be ethanol-induced in both the comparison of ethanol-treated to control and time series ethanol responsive genes (Table S11 in file S2). In addition, *Z. mobilis* responded to ethanol stress primarily by repressing the gene expression at an early stage such as the exponential phase treatment and control comparison and the time-series study of the treated-cells from 6 h to 10 h (Fig. S5 in file S5; Table S9, Table S10 in file S2). For example, 32 genes were down-regulated comparing the treated to the control cells during the exponential phase, which was twice as many as the up-regulated ones (Fig. S5 in file S5, Table S9 in file S2). Time series studies of the ethanol-treated cells’ response from 6 h to 10 h also identified 19 genes down-regulated compared to 2 up-regulated ones only (Fig. S5 in file S5, Table S10 in file S2).

At least 17 genes are likely to be involved in *Z. mobilis* ZM4 hopanoid biosynthesis, including an orphan gene (ZMO1599), four putative operons (ZMO0867-0871; ZMO0874-5; ZMO1547-8; ZMO0972-4) and three additional genes of ZMO0873, ZMO0876, and ZMO0975 adjacent to these operons. In this study, the regulation of hopanoid biosynthetic gene expression was related to the growth phase rather than ethanol treatment. Among the twelve hopanoid biosynthesis proteins identified by proteomic study, none were significantly different between the treatment and control conditions (Table S6 in file S2). Hopanoid amount differences were not observed between treatment and control conditions at the metabolite level either. The transcriptomic study also indicated that ethanol treatment did not significantly change the expression levels of the hopanoid biosynthesis associated genes.

### The Correlation between Proteomic and Transcriptomic Data

Nine hundred and twenty five proteins identified by proteomic study also yielded corresponding gene-expression data, which formed 925 gene-protein pairs (Table S6, Table S7 in file S2). At the same exponential phase as used in the proteomic study, 500 genes were found to be significantly expressed based on JMP Genomics analysis out of the total 925 genes from all the conditions (Table S6 in file S2). The number of significantly expressed genes was more than that of the 95 significantly expressed proteins by the G-test analysis (Table 2, Fig. S5 in file S5, Table S6 in file S2), which is likely due to technical differences between the proteomic and transcriptomic techniques and analyses. Only one protein out of the 95 significantly expressed proteins had no corresponding gene expression data, and there is a correlation coefficient R-squared value (R^2^) of 0.33 between these 94 gene-protein pairs (Table 2, Fig. S5 in file S5, Table S6 in file S2). We can also see a better correlation between proteomic and transcriptomic data for the significantly differentially expressed gene-protein pairs with increasing gene expression differences (Table 2, Fig. S5 in file S5). For example, when we selected the significantly expressed proteins with at least 1.5 or 2-fold change and then compared their correlations to their corresponding genes with significant gene expression, the R^2^ values are 0.49 (49 protein-gene pairs) or 0.50 (40 protein-gene pairs) respectively (Table 2, Fig. S5 in file S5).

**Table 2:** Correlations between log_2_ based expression ratios from transcriptomic and proteomic studies at exponential phase (ethanol-treated cell at 10 h versus control cells at 6 h).

a 1	SP→A	SP1.5→A	SP2.0→A	SP→SA	SP1.5→SA	SP2.0→SA
**Number**	94	84	61	54	49	40
**Correlation**	0.33	0.33	0.33	0.49	0.49	0.50
a **2**	**SA→P**	**SA1.5→P**	**SA2.0→P**	**SA→SP**	**SA1.5→SP**	**SA2.0→SP**
**Number**	500	84	24	54	18	8
**Correlation**	0.17	0.41	0.56	0.49	0.75	0.90
a **3**	**P←→A**			**SP←→SA**	**SP1.5←→SA1.5**	**SA2.0←→SP2.0**
**Number**	925			54	23	8
**Correlation**	0.10			0.49	0.74	0.90

a **:** The direction of comparison; **1**: from significant protein list to identify their corresponding genes for correlation; **2**: from significant gene list to identify their corresponding proteins for correlation; **3**: both the proteins and their corresponding genes used for correlation calculation have same statistical significant differential expression level, and the results of comparisons are same from either directions. **P**: proteomics, **A**: transcriptomics; **S**: statistically significant, **1.5**: at least 1.5-fold difference; **2.0**: at least 2-fold difference. The numbers for proteomic and transcriptomic studies before comparison are: **P**: 942; **SP**: 95 (94 proteins with corresponding gene expression were used for **SP**); **SP1.5**: 84; **SP2.0**: 61; **A**: 1694; **SA**: 912; **SA1.5**: 174; **SA2.0**: 48. **Number**: the number of gene-protein pairs after comparison; **Correlation**: the R-squared number between the log2 based expression ratio (ethanol-treated cell versus control cells) of proteins identified from proteomics and log2 based expression ratio (ethanol-treated cell versus control cells) of genes identified from microarray. **→:** the direction for comparison; For example, **P→A** is to identify the corresponding genes in microarray data from the protein list of proteomic data. **SP1.5→A** is to identify the corresponding genes in microarray data from the protein list of proteomic data with at least 1.5-fold changes.

We identified nine examples where gene expression and protein levels changed in opposite directions, likely pointing to differences in regulation between transcripts and proteins (Table S12 in file S2). For instance, glucose-6-phosphate dehydrogenase gene (ZMO0367), 30S ribosomal protein S5 gene (ZMO0533), and 30S ribosomal protein S6 gene (ZMO1225) had higher expression levels with ethanol treatment (ca 2-fold change), however, the protein level decreased in ethanol treated cells (ca 1.5-fold to 2-fold change). Other example includes six genes such as heat shock protein DnaJ domain-containing gene (ZMO1690), protease ClpA gene (ZMO0405), short-chain dehydrogenase/reductase SDR gene (ZMO0318), alcohol dehydrogenase (ZMO1236) etc., which had a reduced gene expression (ca 1.6-fold to more than 2-fold change) but an increased protein expression (ca 1.6- to more than 8-fold change) levels with the ethanol treatment (Table S12 in file S2).

### ZM4 Operon Predictions and Adjustments

The *Z. mobilis* ZM4 genome annotation has been updated recently [14] and in this study we examined and improved ZM4 operon models. Operons were predicted by our published method [55], [56], [57], which was ranked as the best available operon prediction program by an independent study [62].

We predicted that 1,808 *Z. mobilis* ZM4 genes are organized into 1,048 transcriptional units, 370 of which are multi-gene operons, which is available at DOOR database [57] This predicted operon map is then refined based on the analysis results of available ethanol and salt stress microarray data with 13.8% of the initial predictions rejected since they are not consistent with the microarray gene expression data, and this level of accuracy is comparable to a previous observation [56]. The new operon predictions consist of 366 multi-gene transcripts and 651 single-gene transcripts (Table S13 in file S2). Compared to the original prediction result of 1,048 transcription units, the addition of microarray data reduced the number of transcription units to 1,017.

## Discussion

*Z. mobilis* synthesizes large amounts of hopanoids (up to 30 mg/g, dry weight), which are members of the triterpenic isoprenoids (C_30_) [63], [64]. Hopanoids have been detected in approximately 30% of bacteria tested and are thought to function as prokaryotic sterol analogues involved in membrane integrity, although there is a wealth of different triterpenic (>20,000) structures and their functions are not fully understood [64]. Differences between ethanol-treated cells and control cells were largely due to growth differences (Table S7 in file S2). The sterol-like hopanoids did not appear to be a major response or difference under the stress conditions used in this study. The lack of hopanoid response to ethanol stress is not unusual [33]; however, hopanoids might still be part of a constitutive component of this organism’s ethanol tolerance. The naturally high levels of hopanoids that *Z. mobilis* produce could potentially be exploited to generate isoprenoids of interest for building block chemical synthesis or upgrading to biofuels. The data from global studies such as these will be useful as future synthetic biology and metabolic engineering studies are contemplated.

Addition of ethanol negatively affected growth, carbon utilization and energy maintenance of *Z. mobilis*, and lead to increased fermentation time and reduced ethanol productivity (Fig. 1), which is consistent with a recent transcriptomic study for *Z. mobilis* under 5% ethanol stress published during the reviewing process of our manuscript [25]. In addition, both studies indicated that ethanol has effects on multiple aspects of cellular metabolism [25]. *Z. mobilis* genes or proteins related to translation, ribosomal biogenesis, and flagellar biosynthesis were down-regulated and those related to energy metabolism and stress response such as chaperones were up-regulated for ethanol treated cells. The majority of the early growth phase down-regulated genes that encode hypothetical proteins formed putative operons or clustered together, while the up-regulated genes were scattered around the genome (Fig. 3). The *Clostridium thermocellum* ethanol shock response suggests that nitrogen metabolism plays an important role in wild-type ethanol resistance by enabling ethanol-stressed cells to bypass the carbon metabolism inhibition [65]. A *C. thermocellum* strain with a mutated bifunctional acetaldehyde-CoA/alcohol dehydrogenase gene (*adhE*) had altered cofactor specificity, which likely affects electron flow in the mutant and taken together this indicates different microorganisms respond to such stress using different strategies.

In this study, we updated operon predictions for *Z. mobilis* and this may aid future metabolic engineering endeavors (Table S13 in file S2), just as a prior study joined two genes into the *nhaA* gene for increased tolerance to sodium acetate when over-expressed [22]. The hypothetical characteristics of most down-regulated genes may lay a direction to fully understand the bacterial genome for stress response mechanisms; the differential expression of the hypothetical proteins also provides a way to annotate these genes for future studies, and the clustering expression of the down-regulated genes provides information on operon prediction optimization and bacterial evolution studies. As an example, four genes (ZMO0929, ZMO0930, ZMO0931, and ZMO0932) belonging to an operon predicted by DOOR were down-regulated at least 2-fold at the exponential phase comparison between ethanol-treatment and control condition, but induced in the stationary phase in time series study. Another gene (ZMO0934) originally assigned to the same operon was also down-regulated too with a similar expression pattern, however, the expression value and pattern are different from above four genes and we updated this as a single gene transcription unit.

Although the above four genes were annotated as hypothetical proteins, NCBI Blast search results indicated that actually this region of the chromosome contains two genes, which are *zliE* and *zliS* activating the expression and secretion of levansucrase as reported by Kondo et al. [66]. *Z. mobilis* possesses three sucrases, an intracellular sucrase SacA as well as two extracellular levansucrases of SacB and SacC, which contribute to sucrose hydrolysis. In this study, the structural gene *sacB* (ZMO0374) encoding beta-fructofuranosidase, which is regulated by ZliE and ZliS, was down-regulated in the exponential phase and then induced at a later stage. A SacB homologue has been suggested to be involved in signal transduction for cell wall and membrane composition change, and SacB levansucrase mutants are known to grow on sucrose medium without levan production and produce higher levels of ethanol [67]. The fine-control of the expression of beta-fructofuranosidase SacB may help *Z. mobilis* tolerate the ethanol stress, although further investigation is still needed.

We have reported that a lactate dehydrogenase gene ZMO1237 was more abundant in the relatively more stressful aerobic condition [23]. In this study, there was more lactate produced in ethanol-treated cells when the cells were entering into stationary phase than that of control condition. Expression of a D-lactate dehydrogenase (ZMO0256) was up-regulated in the ethanol-treated cell and it was also induced within the stationary phase. Up-regulation of lactate dehydrogenase genes ZMO1237 and ZMO0256 may lead to the accumulation of lactate in *Z. mobilis*. The production of lactate may help *Z. mobilis* rebalance its reducing power through NADH biosynthesis. It will be useful in the future to compare different stressors or conduct studies where cells are shocked with different inhibitors and their responses immediately assayed and followed over time. An important aspect of this study is that we have confirmed the expression of a large number of *Z. mobilis* proteins for the first time. Likewise, we have added information on transcription levels using a whole genome microarray and this information could be useful for others choosing appropriate promoters to use for metabolic engineering and synthetic biology studies.

Glycerol is a by-product of biodiesel and a potentially abundant and inexpensive source of reducing equivalents [68]. An increase in glycerol flux has been suggested as being important way that *S. cerevisiae* balances intracellular NAD(+)/NADH pools under ethanol stress conditions [69] and redox is evidently important for *C. thermocellum* ethanol tolerance [70]. Exogenous glycerol has been shown to enhance ethanol production from a *Bacillus* species, which was suggested to occur by the presence of additional NADH generated from glycerol uptake and its utilization through glycolysis [71]. In *Z mobilis* growth studies the addition of glycerol to the medium appeared to have a marginal effect on ethanol stress (Fig. S2 in file S5). Further study is required into *Z. mobilis* intracellular redox balance, to define better any role for glycerol and other metabolites in overcoming ethanol stress and to improve the strains performance under industrial conditions. The elimination of undesirable end-products such as lactate or acetate by metabolic engineering of *Z. mobilis* is another avenue for future studies.

We have made a cursory examination of correlation between gene expression and protein levels. We observe better correlations between proteomic and transcriptomic data for the significantly differentially expressed gene-protein pairs with increasing gene expression differences (Table 1, 2, Fig. S5 in file S5). We examined the correlations between the gene or protein expression ratios and abundance, as well as the factors affecting the correlations in this work, rather than investigating the relationship between the gene or protein abundance with the genetic structural characteristics such as codon bias, CDS and RNA secondary structures as reported previously [72], [73]. The present datasets are a rich source for further studies that may address various factors such as RNA secondary structures in transcript and protein comparisons.

Finally, our study has provided insights into the molecular responses of the model ethanologenic bacterium *Z. mobilis* to ethanol stress through an integrated transcriptomic, proteomic, and metabolomic approach, in conjunction with bioinformatics analysis, for the first time. This study provides data on the dynamic levels of *Z. mobilis* molecular responses at a global level, whose better understanding will be requisite to build better models of cellular physiology, its regulation and for manipulation purposes.

## Supporting Information

File S1 **Table S1:** **qPCR primers used in this study.** The information about the primers used for qPCR to verify the microarray results.
(DOCX)

File S2 **Table S2:** **Peptide sequences identified from tandem mass spectrometry measurements.** Proteomics data. **Table S3: List of 30 proteins identified by proteomics that contain non-unique peptides.** Proteomics data. **Table S6: 942 proteins identified from proteomic study for exponential phase** ***Z. mobilis*** **cells.** *Z. mobilis* proteins identified and differences between treatment and control. **Table S7: 1694 genes significantly differentially expressed from treatment versus control and time course studies of control and treatment.** DNA microarray data. **Table S8:** **483 significantly differentially expressed genes comparing ethanol-treated cell versus control cells at different time.** Subset of DNA microarray data. **Table S9:** **571 significantly differentially expressed genes for ZM4 ethanol-treated cells time-series study.** Subset of DNA microarray data. **Table S10: 112 significantly differentially expressed genes for ZM4 ethanol-treated cells time-series study excluding the growth-phase related genes.** Subset of DNA microarray data. **Table S11: 34 common ethanol-responsive genes from both the treatment versus control and time-series studies.** Subset of DNA microarray data. **Table S12: The list of 28 proteins with both proteins and their corresponding genes having at least 1.5-fold statistically significant differences between treatment and control conditions.** Comparison of array and proteomics data. **Table S13: Operon prediction result after adjustment based on microarray results.** Operon IDs after adjustment based on transcriptomic data.
(XLSX)

File S3 **Table S4: The concentrations of extracellular metabolites in ethanol-treated and control cells of** ***Z. mobilis*** **at different time points post-inoculation.** Ethanol supplemented for ethanol treatment is 47 g/L (equal to 6% [v/v]). The concentration units of glucose, ethanol, lactate, acetate and succinate were g/L. Ethanol concentration is the net production amount. ND: non-detectable.
(DOCX)

File S4 **Table S5: Time-course metabolite intracellular concentrations of** ***Zymomonas mobilis*** **ZM4 cultured in control media and media supplemented with 6% ethanol (EtOH).** The average (top value) and standard error of the mean (bottom value) of 2 biological replicate cultures are shown for each treatment at each time point. Concentrations are shown as µg/g fresh weight. Ratios of the metabolite responses of ethanol-treated versus control (top value) and the P-values of Student’s *t*-tests (bottom value) are shown at the right.
(DOCX)

File S5 **Fig. S1:** **The effect of different ethanol concentrations on** ***Z. mobilis*** **growth.** ZM4 growth at 30°C under anaerobic in rich media (RM) broth with or without the supplementation of 16, 32, 47, 63, 79 or 118 g/L ethanol respectively. **Fig. S2: Growth curves for** ***Z. mobilis*** **cultures under ethanol stress and ammended with glycerol.** *Z. mobilis* growth at 30°C under anaerobic conditions. The mean A_600_± S.E. (bars) for 3 replicate growth experiments is shown, with each experiment having at least three independent replicates. **Fig. S3: Molecular weight (MW) and pI distribution of proteins identified through proteomics.** The observed MW and pI distributions for 942 proteins are similar to the theoretical distributions based on genome prediction. **Fig. S4: DNA microarray quality assessments.** Correlation coefficients for biological replicates (**A**), hierarchical clustering analysis based on the correlations (**B**) and Principal Components Analysis (**C**). Treatment arrays are shown in red and control arrays are colored blue. **Fig. S5: Flowchart of metabolite, protein, and gene numbers**. The among metabolome (light yellow boxes), proteome (pale blue boxes), transcriptome (pink box) of ethanol treatment versus control (rose boxes) or time course studies of ethanol-treated cells (purple boxes) and control cells(grey boxes), and different comparisons used in this study to investigate their connections. The boxes drawn with dashed lines indicate the comparison across different omics platforms of metabolomics, proteomics and transcriptomics. Samples were taken at 6, 10, 13.5 or 26 h post-inoculation. Three phase comparisons of ethanol treated versus control cells at exponential phase (**EP**), early stationary phase (**ESP**) and late stationary phase (**LSP**) for metabolomic and transcriptomic studies as well as an exponential phase proteomic comparison study were conducted. **SM**: significant metabolites; **SM1.5**: significant metabolites with at least 1.5-fold changes; **SM2.0**: significant metabolites with at least 2-fold changes; **P**: all the proteins identified from proteomics; **SP**: proteins with significant changes based on G-test result; **SP1.5**: significant proteins with at least 1.5-fold changes; **SP2.0**: significant proteins with at least 2-fold changes; **A**: all the genes identified from transcriptomics; **SA**: significant genes identified from transcriptomics; **SA1.5**: significant genes with at least 1.5-fold changes; **SA2.0**: significant genes with at least 2-fold changes. The numbers after above symbols are the total number identified, and the numbers underneath the symbols are ethanol up-regulated with red font followed by ethanol down-regulated with blue font. The arrows indicate connection between each omics comparison, the orientation of arrow indicates the comparison direction.
(DOCX)
