# Correction: Systems Biology Analysis of *Zymomonas mobilis* ZM4 Ethanol Stress Responses

**DOI:** 10.1371/journal.pone.0101305

**Published:** 2014-06-19

**Authors:** The PLOS ONE Staff

## Notice of Republication

This article was republished on June 2, 2014, to replace File S3 and File S4, which were accidental duplications of File S1. The publisher apologizes for the error. Please view this article again to download the correct files.
