# Supplementary material for: Systems Biology Analysis of Zymomonas mobilis ZM4 Ethanol Stress Responses
Source: PLoS One. 2013 Jul 16;8(7):e68886. doi: 10.1371/journal.pone.0068886 (PMC3712917; doi:10.1371/journal.pone.0068886)
Supplement: File S1 — Table S1: qPCR primers used in this study. The information about the primers used for qPCR to verify the microarray results. (DOCX) [file pone.0068886.s001.docx]

**Table S1.** qPCR Primers used to confirm the transcriptomic study.

| **Primer ID** | **Sequence (written 5' - 3')** |
| --- | --- |
| ZMO0256_qF | GCGGTGCGACTTGATACTTT |
| ZMO0256_qR | CGAAGCTCGGTTAATTCATCA |
| ZMO0367_qF | ACAGCCTGATGAAACCATCC |
| ZMO0367_qR | AACACATCCGTGAGGGAAAG |
| ZMO0369_qF | GCGTTTCTCTATTGCGGAAG |
| ZMO0369_qR | CGAAACGTTCCCAAGCTAAC |
| ZMO0374_qF | AAGAGGAAATTGGCCCTGTT |
| ZMO0374_qR | TTAAGGGCCTGTGCAATACC |
| ZMO0387_qF | GGTAAGGCGCGGCTTTATAC |
| ZMO0387_qR | TCACCATATTTGGCTGACCA |
| ZMO0998_qF | ATCGGCGTTCAATATCGTTC |
| ZMO0998_qR | TTACTATGGGCTTGCGCTTT |
| ZMO1060_qF | AGAAACTGGCTGGTCTGAGC |
| ZMO1060_qR | TTATGATTCCATGCCTGTGC |
| ZMO1236_qF | GTGCCAGATGGTCTTGATCC |
| ZMO1236_qR | GTCCCGGCTGTATCTGAGAA |
| ZMO1576_qF | TTTCCCCGCTTGAGAAACTA |
| ZMO1576_qR | GGCTGCAATCCCATAAAGAA |
| ZMO1622_qF | AGACCAACGCGGACGTATTA |
| ZMO1622_qR | TTCGGACTGTTATCGGGAAG |
