# Supplementary material for: Systems Biology Analysis of Zymomonas mobilis ZM4 Ethanol Stress Responses
Source: PLoS One. 2013 Jul 16;8(7):e68886. doi: 10.1371/journal.pone.0068886 (PMC3712917; doi:10.1371/journal.pone.0068886)
Supplement: File S3 — Table S4: The concentrations of extracellular metabolites in ethanol-treated and control cells of Z. mobilis at different time points post-inoculation. Ethanol supplemented for ethanol treatment is 47 g/L (equal to 6% [v/v]). The concentration units of glucose, ethanol, lactate, acetate and succinate were g/L. Ethanol concentration is the net production amount. ND: non-detectable. (DOCX) [file pone.0068886.s003.docx]

**Table S4.** The concentrations of extracellular metabolites in ethanol-treated and control cells of *Z. mobilis* at different time points post-inoculation. Ethanol supplemented for ethanol treatment is 47 g/L (equal to 6% [v/v]). The concentration units of glucose, ethanol, lactate, acetate and succinate were g/L. Ethanol concentration is the net production amount. ND: not detected.

| *Z. mobilis* in control condition | | | | | |
| --- | --- | --- | --- | --- | --- |
| **Time** | **Glucose** | **Ethanol** | **Lactate** | **Acetate** | **Succinate** |
| **0** | 17.68±0.46 | 0.20±0.01 | 0.023±0.003 | 0.034±0.001 | 0.06±0.002 |
| **2** | 16.32±0.19 | 0.40±0.02 | 0.024±0.002 | 0.037±0.001 | 0.06±0.004 |
| **4** | 15.54±0.46 | 0.96±0.04 | 0.026±0 | 0.039±0.001 | 0.07±0.001 |
| **6** | 11.86±0.10 | 2.44±0.14 | 0.027±0.001 | 0.044±0.001 | 0.09±0.001 |
| **8** | 4.26±1.53 | 4.78±1.12 | 0.026±0.007 | 0.044±0.011 | 0.12±0.008 |
| **10** | 0.05±0.06 | 8.27±0.14 | 0.037±0.001 | 0.073±0.005 | 0.14±0.03 |
| **12** | ND | 8.29±0.08 | 0.037±0.001 | 0.092±0.001 | 0.13±0.006 |
| **14.5** | ND | 8.28±0.06 | 0.038±0 | 0.095±0.007 | 0.13±0.002 |
| **22** | ND | 6.75±1.96 | 0.032±0.008 | 0.097±0.029 | 0.105±0.034 |
| **24** | ND | 7.58±0.58 | 0.034±0.004 | 0.114±0.006 | 0.11±0.018 |
| **26** | ND | 6.77±1.80 | 0.037±0.001 | 0.121±0.002 | 0.12±0.001 |
|  |  |  |  |  |  |
| *Z. mobilis* in 47 g/L ethanol treatment condition | | | | | |
| **Time** | **Glucose** | **Ethanol** | **Lactate** | **Acetate** | **Succinate** |
| **0** | 17.06±0.21 | 0.176±1.83 | 0.023±0 | 0.032±0.005 | 0.06±0.001 |
| **2** | 17.12±0.10 | 0.177±1.56 | 0.026±0 | 0.038±0.004 | 0.06±0.001 |
| **4** | 16.34±0.19 | -0.26±2.24 | 0.025±0 | 0.034±0.004 | 0.06±0.001 |
| **6** | 15.91±0.25 | 0.36±1.93 | 0.026±0 | 0.034±0.005 | 0.06±0.001 |
| **8** | 14.79±0.18 | 0.74±1.53 | 0.028±0.001 | 0.037±0.006 | 0.07±0.005 |
| **10** | 12.14±0.39 | 0.97±2.53 | 0.029±0.001 | 0.039±0.004 | 0.08±0.004 |
| **12** | 7.45±0.31 | 3.80±1.23 | 0.035±0.001 | 0.043±0.001 | 0.11±0.004 |
| **14.5** | 0.056±0.049 | 4.56±2.28 | 0.046±0.001 | 0.051±0.001 | 0.14±0.003 |
| **22** | ND | 5.47±1.37 | 0.045±0.001 | 0.057±0.004 | 0.13±0.004 |
| **24** | ND | 4.27±0.79 | 0.045±0.001 | 0.054±0.001 | 0.13±0.009 |
| **26** | ND | 6.10±1.10 | 0.045±0.001 | 0.054±0.004 | 0.13±0.009 |
