# Supplementary material for: Systems Biology Analysis of Zymomonas mobilis ZM4 Ethanol Stress Responses
Source: PLoS One. 2013 Jul 16;8(7):e68886. doi: 10.1371/journal.pone.0068886 (PMC3712917; doi:10.1371/journal.pone.0068886)
Supplement: File S4 — Table S5: Time-course metabolite intracellular concentrations of Zymomonas mobilis ZM4 cultured in control media and media supplemented with 6% ethanol (EtOH). The average (top value) and standard error of the mean (bottom value) of 2 biological replicate cultures are shown for each treatment at each time point. Concentrations are shown as µg/g fresh weight. Ratios of the metabolite responses of ethanol-treated versus control (top value) and the P-values of Student’s t-tests (bottom value) are shown at the right. (DOCX) [file pone.0068886.s004.docx]

Supplemental Table 5. Time-course metabolite intracellular concentrations (µg/g fresh weight) of *Zymomonas mobilis* ’ZM4’ cultured in control media and media supplemented with 6% ethanol (EtOH). The average (top value) and standard error of the mean (bottom value) of 2 biological replicate cultures are shown for each treatment at each time point. The ratios of the metabolite responses of ethanol-treated versus control (top value) and the P-values of Student’s *t*-tests (bottom value) are shown at the right.

| Metabolite | Control | EtOH | Control | EtOH | Control | EtOH | Control | EtOH | Control | EtOH | Ratio of EtOH/Control | | | | |
| --- | --- | --- | --- | --- | --- | --- | --- | --- | --- | --- | --- | --- | --- | --- | --- |
|  | 6 h | 6 h | 10 h | 10 h | 12 h | 12 h | 13.5 h | 13.5 h | 26 h | 26 h | 6 h | 10 h | 12 h | 13.5 h | 26 h |
| Galactose | 24 | 1023 | 4 | 28 | 6 | 3 | 6 | 5 | 7 | 5 | 43.4 | 6.61 | 0.56 | 0.76 | 0.76 |
|  | 12 | 432 | 1 | 15 | 1 | 0 | 0 | 1 | 1 | 2 | 0.15 | 0.27 | 0.12 | 0.24 | 0.56 |
| Glucose | 530 | 13188 | 9 | 1171 | 58 | 94 | 19 | 24 | 21 | 11 | 24.9 | 137.3 | 1.63 | 1.26 | 0.50 |
|  | 309 | 2698 | 4 | 749 | 44 | 26 | 4 | 6 | 0 | 5 | 0.04 | 0.26 | 0.55 | 0.56 | 0.17 |
| Gulonic acid | 11 | 262 | 31 | 7 | 38 | 6 | 26 | 17 | 29 | 60 | 24.1 | 0.22 | 0.16 | 0.63 | 2.11 |
|  | 1 | 69 | 3 | 0 | 11 | 1 | 1 | 1 | 4 | 34 | 0.07 | 0.02 | 0.11 | 0.04 | 0.45 |
| Fructose | 7 | 122 | 3 | 8 | 6 | 4 | 7 | 6 | 9 | 11 | 17.9 | 2.92 | 0.67 | 0.95 | 1.20 |
|  | 1 | 67 | 1 | 3 | 1 | 1 | 0 | 2 | 0 | 3 | 0.23 | 0.18 | 0.33 | 0.88 | 0.58 |
| Serine | 12 | 105 | 14 | 38 | 36 | 31 | 53 | 43 | 43 | 87 | 9.08 | 2.76 | 0.87 | 0.80 | 2.03 |
|  | 1 | 47 | 0 | 9 | 2 | 8 | 4 | 2 | 4 | 2 | 0.18 | 0.11 | 0.62 | 0.12 | 0.01 |
| Leucine | 33 | 251 | 100 | 149 | 205 | 45 | 166 | 178 | 165 | 65 | 7.55 | 1.49 | 0.22 | 1.07 | 0.40 |
|  | 9 | 205 | 47 | 17 | 24 | 2 | 24 | 58 | 77 | 28 | 0.40 | 0.43 | 0.02 | 0.87 | 0.35 |
| Adenine | 2 | 14 | 23 | 2 | 1 | 1 | 2 | 2 | 1 | 1 | 7.48 | 0.10 | 1.01 | 1.04 | 0.59 |
|  | 0 | 4 | 21 | 0 | 1 | 0 | 1 | 1 | 1 | 0 | 0.10 | 0.44 | 0.98 | 0.97 | 0.49 |
| Ornithine | 6 | 47 | 16 | 10 | 18 | 13 | 26 | 18 | 51 | 31 | 7.37 | 0.65 | 0.73 | 0.70 | 0.61 |
|  | 0 | 29 | 5 | 0 | 1 | 2 | 3 | 4 | 3 | 9 | 0.30 | 0.39 | 0.15 | 0.28 | 0.16 |
| Isoleucine | 32 | 160 | 86 | 136 | 137 | 58 | 125 | 240 | 114 | 66 | 5.06 | 1.57 | 0.43 | 1.92 | 0.58 |
|  | 5 | 117 | 34 | 22 | 19 | 7 | 23 | 52 | 48 | 27 | 0.39 | 0.35 | 0.06 | 0.18 | 0.47 |
| Gluconic acid | 35 | 134 | 1 | 74 | 4 | 52 | 4 | 6 | 5 | 4 | 3.78 | 55.9 | 13.92 | 1.42 | 0.77 |
|  | 10 | 55 | 0 | 6 | 1 | 1 | 0 | 1 | 0 | 0 | 0.22 | 0.01 | 0.00 | 0.17 | 0.15 |
| Trehalose | 417 | 1535 | 350 | 489 | 529 | 367 | 509 | 649 | 673 | 1260 | 3.68 | 1.40 | 0.69 | 1.27 | 1.87 |
|  | 33 | 781 | 42 | 26 | 76 | 21 | 41 | 131 | 131 | 689 | 0.29 | 0.11 | 0.18 | 0.42 | 0.49 |
| Lysine | 1 | 5 | 10 | 3 | 2 | 6 | 7 | 9 | 11 | 8 | 3.66 | 0.32 | 2.73 | 1.35 | 0.76 |
|  | 1 | 2 | 8 | 2 | 2 | 5 | 2 | 3 | 1 | 1 | 0.25 | 0.48 | 0.54 | 0.57 | 0.17 |
| Threonine | 18 | 64 | 21 | 30 | 39 | 28 | 54 | 46 | 61 | 53 | 3.55 | 1.44 | 0.74 | 0.86 | 0.86 |
|  | 2 | 28 | 1 | 6 | 5 | 6 | 5 | 2 | 6 | 1 | 0.24 | 0.27 | 0.34 | 0.31 | 0.31 |
| Aspartic acid | 129 | 452 | 21 | 208 | 7 | 135 | 3 | 389 | 3 | 2 | 3.51 | 9.86 | 19.28 | 123.4 | 0.87 |
|  | 92 | 185 | 16 | 60 | 3 | 6 | 0 | 0 | 0 | 0 | 0.26 | 0.10 | 0.00 | 0.00 | 0.44 |
| Myoinositol | 3 | 11 | 3 | 4 | 4 | 3 | 5 | 5 | 6 | 5 | 3.46 | 1.29 | 0.72 | 0.95 | 0.78 |
|  | 0 | 4 | 1 | 0 | 0 | 0 | 0 | 1 | 1 | 1 | 0.23 | 0.28 | 0.05 | 0.78 | 0.38 |
| Valine | 131 | 372 | 158 | 397 | 286 | 134 | 195 | 528 | 256 | 105 | 2.85 | 2.51 | 0.47 | 2.71 | 0.41 |
|  | 4 | 266 | 97 | 61 | 47 | 13 | 18 | 262 | 113 | 27 | 0.46 | 0.17 | 0.09 | 0.33 | 0.32 |
| Glycerol | 71 | 188 | 152 | 159 | 198 | 151 | 234 | 397 | 252 | 551 | 2.66 | 1.05 | 0.76 | 1.70 | 2.19 |
|  | 2 | 78 | 52 | 18 | 50 | 15 | 27 | 62 | 48 | 327 | 0.27 | 0.90 | 0.46 | 0.14 | 0.46 |
| Malic acid | 3 | 9 | 2 | 4 | 3 | 3 | 3 | 5 | 3 | 3 | 2.60 | 2.09 | 1.36 | 1.99 | 0.98 |
|  | 0 | 3 | 0 | 0 | 1 | 0 | 0 | 1 | 1 | 0 | 0.19 | 0.06 | 0.29 | 0.07 | 0.95 |
| Phenylalanine | 146 | 364 | 168 | 192 | 243 | 168 | 335 | 325 | 344 | 334 | 2.48 | 1.14 | 0.69 | 0.97 | 0.97 |
|  | 19 | 157 | 19 | 11 | 48 | 6 | 2 | 62 | 12 | 23 | 0.31 | 0.40 | 0.26 | 0.89 | 0.73 |
| Alanine | 151 | 338 | 187 | 194 | 261 | 106 | 273 | 188 | 297 | 136 | 2.24 | 1.03 | 0.41 | 0.69 | 0.46 |
|  | 21 | 186 | 87 | 15 | 45 | 5 | 16 | 188 | 58 | 83 | 0.42 | 0.95 | 0.07 | 0.70 | 0.25 |
| Asparagine | 6 | 13 | 5 | 7 | 6 | 5 | 10 | 9 | 9 | 10 | 2.23 | 1.30 | 0.91 | 0.94 | 1.07 |
|  | 1 | 2 | 1 | 1 | 1 | 1 | 0 | 1 | 1 | 0 | 0.10 | 0.37 | 0.68 | 0.41 | 0.41 |
| Glucose 6-P | 8 | 17 | 7 | 9 | 3 | 4 | 4 | 2 | 4 | 2 | 2.17 | 1.35 | 1.23 | 0.43 | 0.56 |
|  | 0 | 3 | 4 | 1 | 1 | 1 | 0 | 0 | 0 | 0 | 0.12 | 0.60 | 0.59 | 0.03 | 0.01 |
| Fumaric acid | 3 | 5 | 2 | 3 | 3 | 3 | 4 | 5 | 5 | 5 | 2.04 | 1.63 | 0.98 | 1.28 | 0.86 |
|  | 0 | 1 | 0 | 0 | 0 | 0 | 0 | 1 | 1 | 1 | 0.16 | 0.12 | 0.88 | 0.31 | 0.58 |
| 5-oxo-Proline | 512 | 986 | 322 | 692 | 334 | 498 | 466 | 864 | 436 | 261 | 1.92 | 2.15 | 1.49 | 1.85 | 0.60 |
|  | 36 | 247 | 93 | 78 | 60 | 9 | 4 | 80 | 6 | 132 | 0.20 | 0.09 | 0.11 | 0.04 | 0.32 |
| Erythronic acid | 1 | 1 | 1 | 1 | 1 | 1 | 2 | 1 | 1 | 1 | 1.90 | 1.04 | 0.63 | 0.80 | 0.52 |
|  | 0 | 1 | 0 | 0 | 0 | 0 | 0 | 0 | 0 | 0 | 0.36 | 0.81 | 0.32 | 0.33 | 0.04 |
| Glutamic acid | 414 | 785 | 184 | 545 | 85 | 356 | 104 | 848 | 125 | 89 | 1.90 | 2.96 | 4.19 | 8.12 | 0.71 |
|  | 55 | 209 | 123 | 76 | 17 | 4 | 4 | 83 | 6 | 0 | 0.23 | 0.13 | 0.00 | 0.01 | 0.03 |
| Citric acid | 5 | 9 | 2 | 4 | 1 | 3 | 2 | 3 | 2 | 1 | 1.85 | 2.49 | 2.28 | 1.80 | 0.82 |
|  | 0 | 2 | 0 | 1 | 0 | 0 | 0 | 0 | 0 | 0 | 0.19 | 0.13 | 0.03 | 0.10 | 0.58 |
| Tryptophan | 2 | 3 | 24 | 19 | 116 | 18 | 92 | 3 | 136 | 4 | 1.69 | 0.82 | 0.15 | 0.03 | 0.03 |
|  | 1 | 0 | 2 | 0 | 2 | 0 | 1 | 3 | 1 | 1 | 0.57 | 0.89 | 0.17 | 0.14 | 0.00 |
| Phosphate | 1533 | 2564 | 1699 | 1897 | 2463 | 1627 | 3820 | 2747 | 3442 | 2137 | 1.67 | 1.12 | 0.66 | 0.72 | 0.62 |
|  | 204 | 559 | 276 | 43 | 315 | 125 | 161 | 448 | 222 | 1099 | 0.23 | 0.55 | 0.13 | 0.15 | 0.36 |
| Myristic acid | 2 | 4 | 2 | 2 | 2 | 2 | 3 | 3 | 4 | 3 | 1.51 | 1.57 | 0.98 | 0.92 | 0.86 |
|  | 0 | 0 | 0 | 0 | 0 | 0 | 0 | 0 | 0 | 0 | 0.04 | 0.18 | 0.75 | 0.74 | 0.21 |
| Glycine | 473 | 694 | 425 | 584 | 480 | 523 | 647 | 1162 | 654 | 520 | 1.47 | 1.37 | 1.09 | 1.80 | 0.80 |
|  | 32 | 167 | 88 | 56 | 107 | 24 | 26 | 97 | 26 | 121 | 0.32 | 0.27 | 0.73 | 0.04 | 0.39 |
| Succinic acid | 58 | 75 | 60 | 69 | 62 | 71 | 75 | 148 | 70 | 111 | 1.29 | 1.15 | 1.15 | 1.97 | 1.58 |
|  | 7 | 22 | 5 | 5 | 17 | 1 | 2 | 12 | 6 | 14 | 0.53 | 0.30 | 0.64 | 0.03 | 0.12 |
| Proline | 3 | 3 | 1 | 2 | 2 | 2 | 3 | 2 | 3 | 2 | 1.19 | 1.73 | 1.05 | 0.93 | 0.63 |
|  | 0 | 0 | 0 | 0 | 0 | 0 | 0 | 1 | 0 | 1 | 0.18 | 0.07 | 0.76 | 0.80 | 0.37 |
| Adenosine | 44 | 52 | 12 | 23 | 20 | 15 | 23 | 17 | 21 | 8 | 1.18 | 1.96 | 0.74 | 0.74 | 0.39 |
|  | 9 | 18 | 4 | 1 | 1 | 1 | 5 | 5 | 4 | 0 | 0.74 | 0.10 | 0.06 | 0.49 | 0.09 |
| Palmitic acid | 22 | 26 | 11 | 20 | 16 | 15 | 23 | 22 | 27 | 23 | 1.15 | 1.91 | 0.92 | 0.94 | 0.84 |
|  | 0 | 3 | 3 | 0 | 1 | 1 | 0 | 5 | 3 | 2 | 0.43 | 0.10 | 0.41 | 0.82 | 0.39 |
| Glutamine | 38 | 42 | 34 | 62 | 45 | 47 | 36 | 72 | 73 | 46 | 1.11 | 1.84 | 1.05 | 2.00 | 0.64 |
|  | 24 | 29 | 8 | 1 | 4 | 5 | 30 | 19 | 4 | 12 | 0.92 | 0.07 | 0.77 | 0.42 | 0.17 |
| GABA | 151 | 163 | 53 | 74 | 64 | 144 | 98 | 280 | 113 | 164 | 1.08 | 1.39 | 2.23 | 2.84 | 1.44 |
|  | 27 | 6 | 13 | 6 | 12 | 8 | 3 | 32 | 0 | 35 | 0.69 | 0.28 | 0.03 | 0.03 | 0.29 |
| Stearic acid | 15 | 16 | 6 | 12 | 8 | 8 | 10 | 11 | 11 | 8 | 1.05 | 2.11 | 0.96 | 1.09 | 0.76 |
|  | 1 | 0 | 2 | 0 | 2 | 0 | 1 | 3 | 1 | 1 | 0.35 | 0.09 | 0.86 | 0.77 | 0.26 |
| Thymine | 2 | 1 | 1 | 1 | 1 | 1 | 2 | 2 | 2 | 2 | 0.79 | 1.39 | 0.58 | 0.85 | 1.15 |
|  | 0 | 1 | 0 | 0 | 0 | 0 | 0 | 0 | 0 | 0 | 0.73 | 0.20 | 0.33 | 0.47 | 0.39 |
| Glycerol 1/3-P | 77 | 51 | 63 | 74 | 74 | 70 | 112 | 109 | 102 | 73 | 0.66 | 1.17 | 0.95 | 0.98 | 0.71 |
|  | 5 | 8 | 16 | 4 | 23 | 6 | 11 | 19 | 6 | 2 | 0.12 | 0.60 | 0.89 | 0.92 | 0.05 |
| Glyceric acid | 10 | 6 | 1 | 5 | 1 | 3 | 2 | 2 | 2 | 2 | 0.66 | 4.75 | 2.28 | 1.21 | 0.95 |
|  | 1 | 2 | 0 | 1 | 0 | 1 | 0 | 0 | 0 | 0 | 0.24 | 0.05 | 0.23 | 0.47 | 0.81 |
| Shikimic acid | 96 | 51 | 73 | 89 | 155 | 93 | 192 | 138 | 193 | 206 | 0.53 | 1.22 | 0.60 | 0.72 | 1.07 |
|  | 24 | 11 | 5 | 7 | 30 | 0 | 1 | 23 | 19 | 24 | 0.23 | 0.19 | 0.17 | 0.14 | 0.72 |
| Methionine | 3 | 1 | 1 | 1 | 5 | 1 | 1 | 19 | 1 | 1 | 0.44 | 0.50 | 0.10 | 18.40 | 1.17 |
|  | 1 | 0 | 0 | 0 | 1 | 0 | 0 | 1 | 0 | 0 | 0.22 | 0.21 | 0.02 | 0.00 | 0.61 |
| Ethyl phosphate | 28 | 9 | 21 | 30 | 21 | 33 | 35 | 59 | 27 | 35 | 0.34 | 1.41 | 1.53 | 1.68 | 1.32 |
|  | 2 | 0 | 5 | 4 | 5 | 1 | 1 | 8 | 4 | 15 | 0.01 | 0.28 | 0.16 | 0.09 | 0.63 |
| Oleic acid | 9 | 3 | 12 | 10 | 17 | 13 | 36 | 29 | 47 | 40 | 0.34 | 0.80 | 0.75 | 0.82 | 0.84 |
|  | 0 | 2 | 4 | 0 | 4 | 1 | 1 | 5 | 7 | 2 | 0.08 | 0.58 | 0.39 | 0.32 | 0.39 |
