# Supplementary material for: Systems Biology Analysis of Zymomonas mobilis ZM4 Ethanol Stress Responses
Source: PLoS One. 2013 Jul 16;8(7):e68886. doi: 10.1371/journal.pone.0068886 (PMC3712917; doi:10.1371/journal.pone.0068886)

**Figure S1.** The effect of different ethanol concentrations on *Z. mobilis* growth.

**Figure S2.** Growth curves for *Z. mobilis* cultures under ethanol stress and ammended with glycerol.


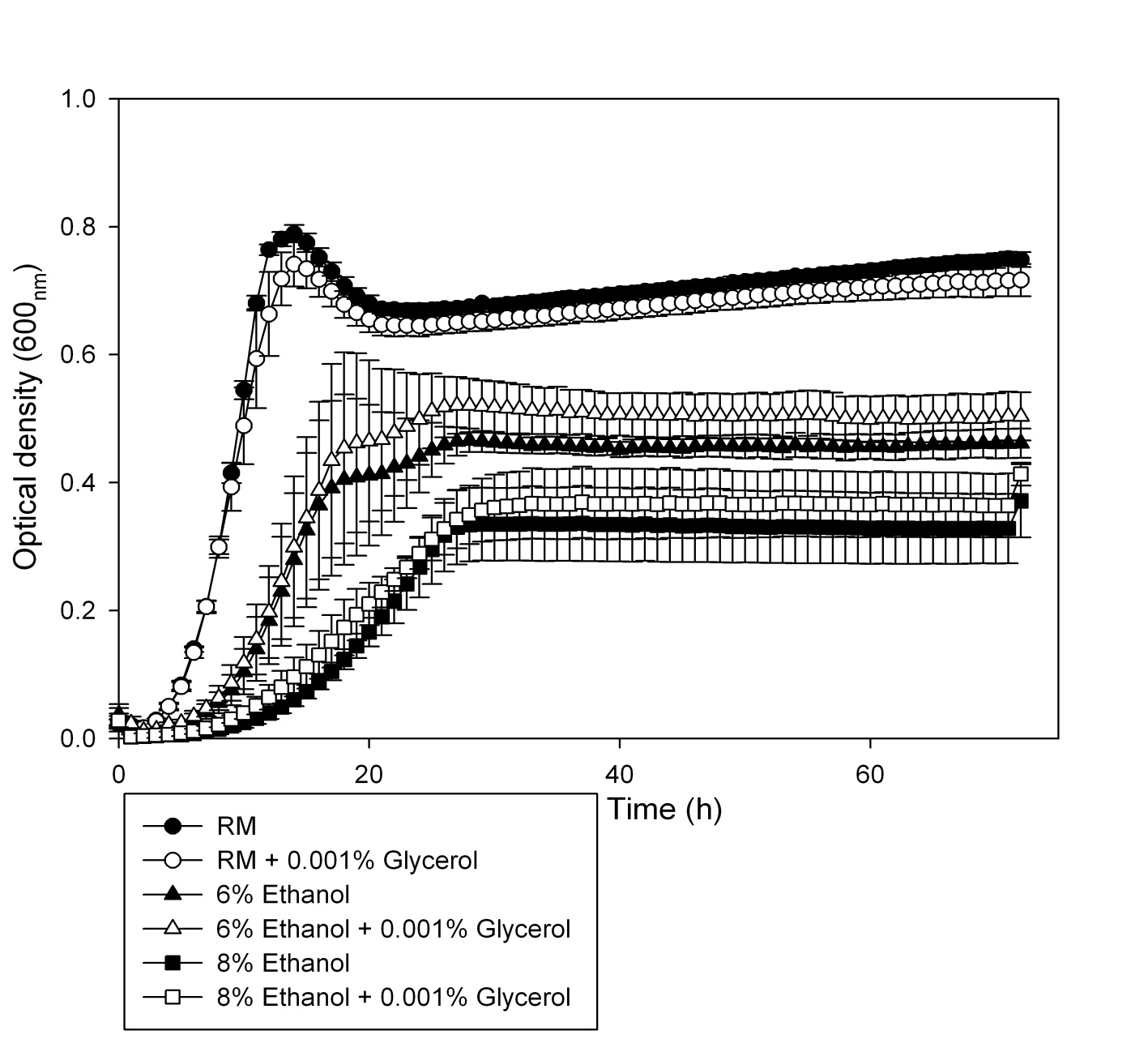


**Figure S3.** Molecular weight (MW) and pI distribution of proteins identified through proteomics. 942 proteins were identified (ca 55% of the total predicted proteins) with most of them are identified in both ethanol treatment and control conditions. The observed MW and pI distributions are similar to the theoretical distributions based on genome prediction.

**Figure S4.** DNA microarray quality assessments. Correlation coefficients for biological replicates (**A**), hierarchical clustering analysis based on the correlations (**B**) and Principal Components Analysis (PCA) (**C**) of microarray data generated in this study. Treatment arrays are shown in red and control arrays are colored blue.

**(A)**

**6h**

**6h**

**10h**

**13.5h**

**26h**

**10, 13.5h**

**26h**

**B**

**C**

**Figure S5.** The flowchart of metabolite, protein, and gene numbers among metabolome (light yellow boxes), proteome (pale blue boxes), transcriptome (pink box) of ethanol treatment versus control (rose boxes) or time course studies of ethanol-treated cells (purple boxes) and control cells(grey boxes), and different comparisons used in this study to investigate their connections. The boxes drawn with dashed lines indicate the comparison across different omics platforms of metabolomics, proteomics and transcriptomics. Samples were taken at 6, 10, 13.5 or 26 h post-inoculation. Three phase comparisons of ethanol treated versus control cells at exponential phase (**EP**), early stationary phase (**ESP**) and late stationary phase (**LSP**) for metabolomic and transcriptomic studies as well as an exponential phase proteomic comparison study were conducted. **SM**: significant metabolites; **SM1.5**: significant metabolites with at least 1.5-fold changes; **SM2.0**: significant metabolites with at least 2-fold changes; **P**: all the proteins identified from proteomics; **SP**: proteins with significant changes based on G-test result; **SP1.5**: significant proteins with at least 1.5-fold changes; **SP2.0**: significant proteins with at least 2-fold changes; **A**: all the genes identified from transcriptomics; **SA**: significant genes identified from transcriptomics; **SA1.5**: significant genes with at least 1.5-fold changes; **SA2.0**: significant genes with at least 2-fold changes. The numbers after above symbols are the total number identified, and the numbers underneath the symbols are ethanol up-regulated with red font followed by ethanol down-regulated with blue font. The arrows indicate connection between each omics comparison, the orientation of arrow indicates the comparison direction.


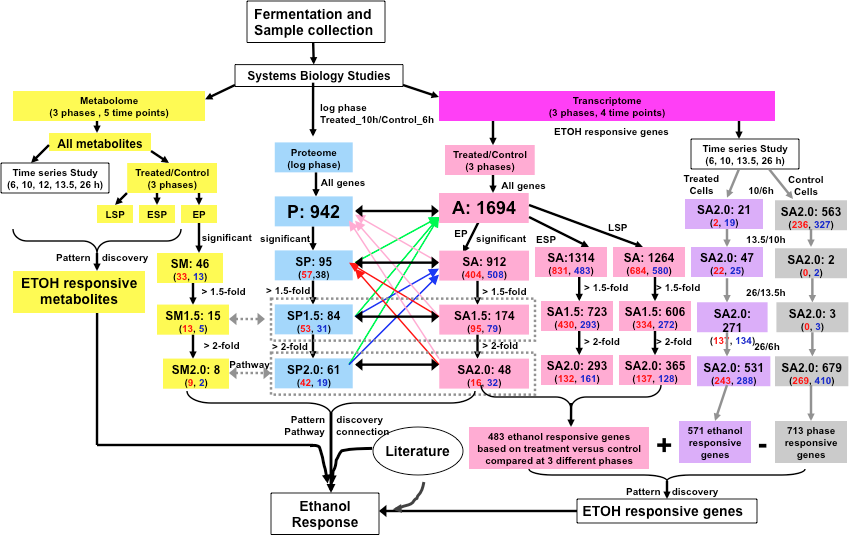

Supplement: File S5 — Fig. S1: The effect of different ethanol concentrations on Z. mobilis growth. ZM4 growth at 30°C under anaerobic in rich media (RM) broth with or without the supplementation of 16, 32, 47, 63, 79 or 118 g/L ethanol respectively. Fig. S2: Growth curves for Z. mobilis cultures under ethanol stress and ammended with glycerol. Z. mobilis growth at 30°C under anaerobic conditions. The mean A600± S.E. (bars) for 3 replicate growth experiments is shown, with each experiment having at least three independent replicates. Fig. S3: Molecular weight (MW) and pI distribution of proteins identified through proteomics. The observed MW and pI distributions for 942 proteins are similar to the theoretical distributions based on genome prediction. Fig. S4: DNA microarray quality assessments. Correlation coefficients for biological replicates (A), hierarchical clustering analysis based on the correlations (B) and Principal Components Analysis (C). Treatment arrays are shown in red and control arrays are colored blue. Fig. S5: Flowchart of metabolite, protein, and gene numbers. The among metabolome (light yellow boxes), proteome (pale blue boxes), transcriptome (pink box) of ethanol treatment versus control (rose boxes) or time course studies of ethanol-treated cells (purple boxes) and control cells(grey boxes), and different comparisons used in this study to investigate their connections. The boxes drawn with dashed lines indicate the comparison across different omics platforms of metabolomics, proteomics and transcriptomics. Samples were taken at 6, 10, 13.5 or 26 h post-inoculation. Three phase comparisons of ethanol treated versus control cells at exponential phase (EP), early stationary phase (ESP) and late stationary phase (LSP) for metabolomic and transcriptomic studies as well as an exponential phase proteomic comparison study were conducted. SM: significant metabolites; SM1.5: significant metabolites with at least 1.5-fold changes; SM2.0: significant metabolites with at least 2 [file pone.0068886.s005.docx]
